# Chemico-pharmacological evaluations of the dwarf elephant ear (*Colocasia affinis* Schott) plant metabolites and extracts: health benefits from vegetable source

**DOI:** 10.3389/fphar.2024.1428341

**Published:** 2024-08-13

**Authors:** Safaet Alam, Fahmida Tasnim Richi, Hasin Hasnat, Firoj Ahmed, Nazim Uddin Emon, Md. Jasim Uddin, G. M. Masud Rana, Shuanghu Wang, Mst. Sarmina Yeasmin, Nazim Uddin Ahmed, Md. Salim Khan, Abdullah Al Mamun

**Affiliations:** ^1^ Chemical Research Division, BCSIR Dhaka Laboratories, Bangladesh Council of Scientific and Industrial Research (BCSIR), Dhaka, Bangladesh; ^2^ Bangladesh Council of Scientific and Industrial Research, Rajshahi, Bangladesh; ^3^ Department of Pharmaceutical Chemistry, Faculty of Pharmacy, University of Dhaka, Dhaka, Bangladesh; ^4^ Department of Pharmacy, State University of Bangladesh, Dhaka, Bangladesh; ^5^ Department of Pharmacy, Faculty of Science and Engineering, International Islamic University Chittagong, Chittagong, Bangladesh; ^6^ Central Laboratory of The Lishui Hospital of Wenzhou Medical University, Lishui People’s Hospital, The First Affiliated Hospital of Lishui University, Lishui, Zhejiang, China

**Keywords:** *Colocasia affinis*, *in vitro*, antioxidant, cytotoxicity, antimicrobial, *in vivo*, antidiarrheal, analgesic

## Abstract

**Introduction:** *Colocasia affinis* Schott (Family: Araceae), found in the Asian region, is a traditional root vegetable consumed by the locals and well-known as Dwarf Elephant Ear.

**Methods:** For the pharmacological exploration of this root vegetable, four kupchan fractions (i.e. HSF, DCMSF, EASF, and AQSF) from ethanolic extract of *C. affinis* were employed to *in vitro* i.e. antioxidant, cytotoxicity, and antimicrobial and *in vivo* i.e. antidiarrheal and analgesic assays, followed by phytochemical screening and GC-MS protocol.

**Result and Discussion:** In the antioxidant assay, the AQSF showed promising potential with an IC_50_ value of 29.4 μg/mL and additionally, it exhibited the greatest overall phenolic content, measuring 57.23 mg GAE/gm. of extract among other fractions. The AQSF also revealed promising cytotoxic activity in brine shrimp lethality assay with an LC_50_ value of 1.36 μg/mL. Both AQSF and EASF exhibited substantial antimicrobial efficacy against both gram-positive and gram-negative bacteria as well as various fungus species with a remarkable zone of inhibitions compared to standards. Whereas, during both the castor oil-induced antidiarrheal and acetic acid-induced writhing assay, the DCMSF at 400 mg/kg dose exhibited the highest 51.16% reduction of diarrhea and 52.33% reduction of writhing. Phytochemical screening revealed several chemical groups while GC-MS study of different fractions of dwarf elephant ear ethanolic extract revealed 48 different bioactive phytochemicals in total. Several targets such as KAS, DHFR for anti-microbial activities, GLR, URO for antioxidant activities, EGFR, BCL-2 for cytotoxicity, KOR, DOR for antidiarrheal activities and COX-2, TNF-α for analgesic activities are considered for molecular docking against identified phytocompounds and standards along with ADME/T studies to ascertain their safety, efficacy and drug likeliness profiles.

**Conclusion:** To recapitulate, our study revealed that vegetables such as dwarf elephant ear can be considered as a prospective source of therapeutics and drug development besides their nutritive food values.

## Introduction

Vegetables are essential to the human diet, providing vital vitamins, minerals, nutrients, and bioactive secondary metabolites that support health and enhance food’s color and flavor (Naczk and Shahidi, 2006). Vegetables have been utilized for medical purposes and illness prevention since ancient times. Foods high in antioxidants are in greater demand due to growing health consciousness. Including fresh veggies in the diet is a cheap method to receive a variety of nutrients (Webb and Villamor, 2007). Many vegetables contain immune-boosting macromolecules, carotenoids, flavonoids, and vitamin C. They are also rich in antioxidants like tannins, phenolic acids, flavonoids, and various metabolites (Bhat and Al-Daihan, 2014; Thakur, 2018). Studies have revealed that various antioxidant compounds in vegetables demonstrate diverse activities, including but not limited to anti-inflammatory, antitumor, anticarcinogenic, antimutagenic, anti-atherosclerotic, antibacterial, and antiviral properties (Alonso et al., 2004; Hamzah et al., 2014). Moreover, almost 80% of all commercially available drug substances either directly come from natural metabolites or represent a modified version of the natural analogs (Alam et al., 2021a). Synthetic drugs face safety, efficacy, multi-drug resistance, availability, and cost-effectiveness challenges. Therefore, extracts from vegetable leaves, roots, or whole plants could become valuable sources for natural drug discovery (Maridass and De Britto, 2008; Sudhakar et al., 2020).

Oxidative stress, an imbalance between pro-oxidants and antioxidants, is a major concern linked to chronic illnesses such as diabetes, obesity, metabolic syndrome, cancer, neurological and cardiovascular disorders, as well as inflammatory diseases, with elevated reactive oxygen species (ROS) promoting tumor formation and cancer cell proliferation (Hayes et al., 2020; Morvaridzadeh et al., 2020). With more than 14 million new cases diagnosed every year, cancer ranks high among the world’s leading causes of mortality. By 2030, experts predict that this figure will have almost doubled (Klaunig, 2019). Pain from inflammation arises when the body’s response to injury, infection, or irritation releases signaling molecules like prostaglandins and cytokines, leading to discomfort and adverse effects such as degeneration, necrosis, and exudations (Omoigui, 2007). While NSAIDs, opioids, and non-opioids are effective for managing inflammation and pain (Alam et al., 2020), prolonged use can lead to serious side effects, such as respiratory depression, cardiovascular issues, GI ulceration, liver toxicity, and renal impairment (Carter et al., 2014). Consequently, it is needed to explore and analyze emerging secondary metabolites for better alternatives (Liu, 2007). Diarrhea, marked by frequent, loose bowel movements, is the fifth leading cause of death in developing countries and the eighth worldwide. Despite progress in reducing early childhood deaths, diarrheal morbidity and mortality remain high in older children and adults, with 5.7 billion cases and 1.1 million deaths in 2019. Although most cases are benign, about 285 million annually (5%) result in moderate to severe disease requiring medical treatment (Abbafati et al., 2020; Levine et al., 2023). The underlying causes of diarrhea include unhygienic lifestyles and exposure to pathogenic microbes like bacteria, fungi, viruses, and parasites. *Candida albicans*, *Escherichia coli*, *Salmonella typhi*, *Shigella flexner*i, and *Staphylococcus aureus* are common among the many entero-pathogens that can cause diarrhea (Alam et al., 2021a). Antibiotics and other antimicrobial therapy are no less prevalent in diarrheal management than in other infectious diseases, giving rise to global health emergencies like ‘antibiotic/antimicrobial resistance,’ ‘antibiotic-associated diarrhea,’ ‘multi-drug resistance,’ etc. (Mekonnen et al., 2020; Darby et al., 2023). Therefore, clinical microbiologists are focusing on plant-derived antimicrobials to natural plant extract antimicrobials over conventional antibiotics (Sher, 2009; Tiwari et al., 2023).

*Colocasia affinis* Schott, belonging to the family Araceae, is one of the most prominent species with medicinal food values among the 9 species of the genus *Colocasia* cultivated throughout the northern tropical area of Bangladesh (Alam et al., 2021a). Indigenously, this 46–91 cm tall perennial herb is known as ‘Kochu’ (Bengali) or ‘Dwarf Elephant’s Ear’ (English) and can be found all over Bangladesh, especially, in Chittagong hill tracts and rural areas of the country. It is also available in Thailand, India, Myanmar, and other Asian countries (Mondal et al., 2019). The leaves, leaf stalk, and tuber of this perennial food crop are used as vegetables in curries and stews (Mondal et al., 2019; Dev et al., 2021). The green leafy vegetables of *Colocasia* botanical drugs were also found to be used as savory and a rich source of calcium, iron, vitamin C, and other secondary metabolites, having remarkable potential in diabetes management, hypertension control, immune support, neuroprotection, and anticarcinogenic activities (Gupta et al., 2019). In different traditional medicine systems, fever, wound healing, infection, phlegm, constipation, atrophy, emaciation, drowsiness, tuberculosis, arresting arterial hemorrhage, fading melasma and ameliorating stomach issues were among the conditions for which *Colocasia* botanical drugs have been utilized (Pornprasertpol et al., 2015; Sudhakar et al., 2020). Specifically, *C. affinis* was used by the locals to treat cataracts (Mondal et al., 2019). A research study reported that this edible botanical drug contains different alkaloids, flavonoids, glycosides, saponins, tannins, steroids, triterpenoids, fatty acids derivatives, carbohydrates, proteins, and amino acids (Lalremruati et al., 2018)

Prior studies on *C. affinis* have predominantly concentrated on its nutritional worth and customary application. Nevertheless, evidence is scarce regarding the extensive pharmacological capabilities and chemical characterization of the substance, particularly when employing modern techniques such as GC-MS. Research has indicated that *Colocasia* species possess bioactive substances that offer diverse health advantages, while there is a lack of comprehensive pharmacological assessments. This study will combine *in vitro* and *in vivo* pharmacological assays with rigorous GC-MS analysis and molecular docking to thoroughly examine the bioactive metabolites of *C. affinis* Schott. This research will be groundbreaking in finding precise molecular targets and pharmacological potentials of this traditional vegetable, which has not been thoroughly investigated previously. This comprehensive approach will emphasize the medicinal potential of the subject, going beyond its nutritional benefits.

## Material and methods

### Collection of plant

**FIGURE 1:**
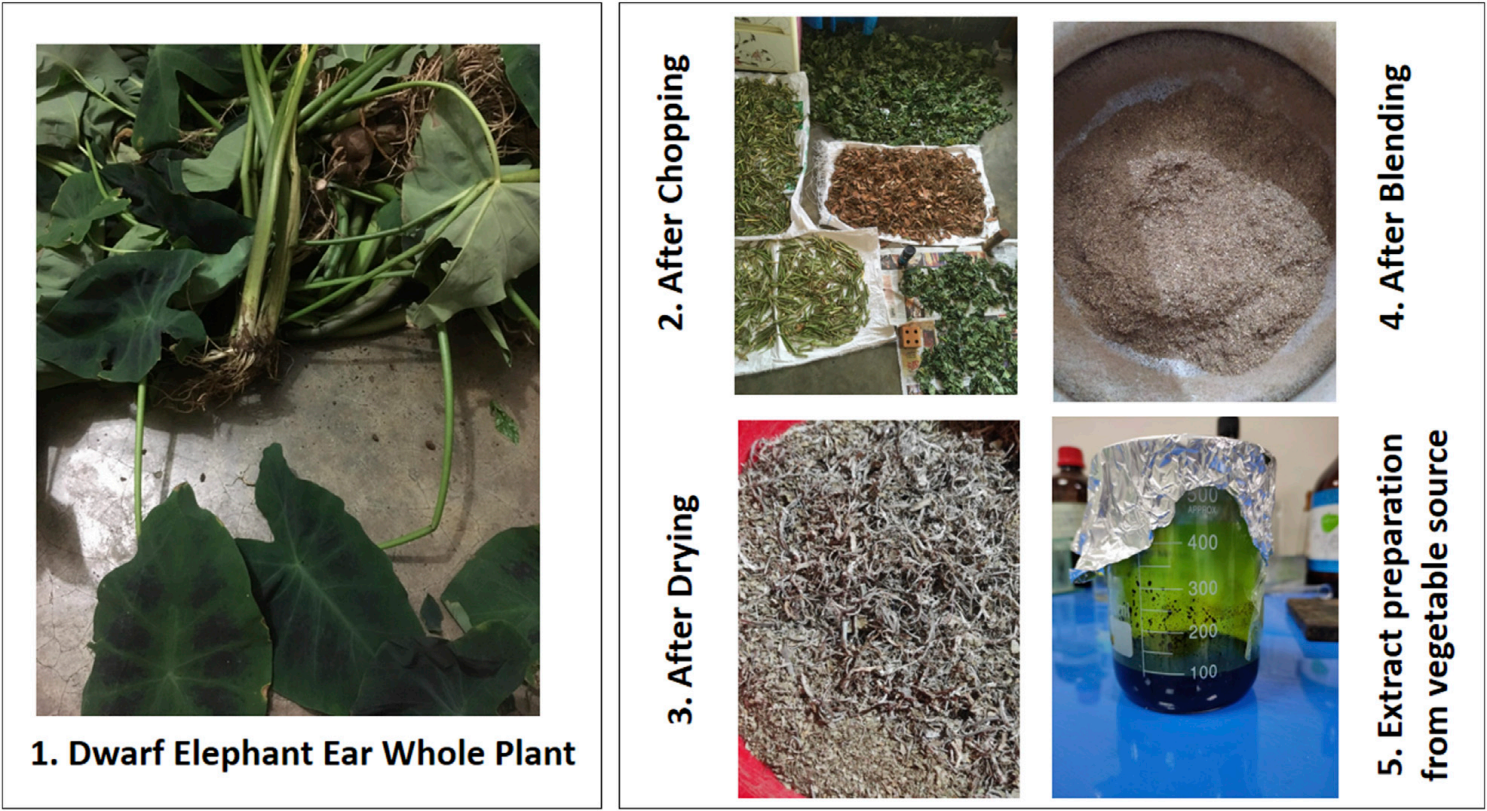
Dwarf elephant ear collection and vegetable extract preparation.

Whole plants of *C. affinis* were picked from the hill areas of Bandarban, Chittagong, Bangladesh, in September 2022 (Figure 1). In Mirpur, Dhaka, the specialists at the Bangladesh National Herbarium confirmed the authenticity of the plant samples. The plant’s voucher specimen, labeled with the accession number 57065, has been stored in the herbarium for forthcoming reference.

### Drug and chemicals

Analytical-grade medicines and substances were employed in this investigation. Ethanol and Tween-80 were bought from Merk (Darmstadt, Germany). Folin-Ciocalteau reagent (FCR) and 1,1-diphenyl -2-picryl-hydroxyl radical (DPPH) were procured from Sigma Chemicals Co. (St. Louis, Missouri, United States). Diclofenac sodium, Azithromycin, Amoxicillin, Ciprofloxacin, and Fluconazole were purchased from the following suppliers, respectively (Square Pharmaceuticals Ltd., Bangladesh, and Incepta Pharmaceuticals Ltd., Bangladesh).

### Test microorganisms

For the antimicrobial assay, gram-positive bacteria (*Sarcina lutea, Bacillus megaterium, S. aureus*, *Bacillus cereus,* and *Bacillus subtilis*), gram-negative bacteria (*Vibrio mimicus*, *Pseudomonas aeruginosa, S. typhi, Salmonella paratyphi, E. coli*, *Shigella dysenteriae,* and *Vibrio parahemolyticus*) and fungi strains (*Aspergillus niger*, *Sacharomyces cerevacae,* and *C. albicans*) were utilized, provided from University of Dhaka, Bangladesh.

### Experimental design

#### Extraction of plant materials

The whole plant of *C. affinis* was thoroughly washed and then sun-dried. After drying the moisture content was around 10%. Afterward, a grinding apparatus was used to process the dried plant into a coarse powder. Then 800 gm of dried leaf power was placed into a 5L round-bottomed clean flask. This 2.5L of ethanol (analytical grade, with ≥96% purity) was added and kept for 21 days with periodic shaking and stirring. After that, the entire mixture (32% w/v) was filtered using Whatman No.1 filter paper, followed by a new cotton plug. The method was repeated thrice with analytical-grade ethanol. Buchi Rotavapor was then used to decrease the filtrate content at low temperatures and pressures. The crude extract weighed 60.82 g (7.57% yield value).

#### Partition into different fractionated extractives

The Kupchan fractionation method was employed to fractionate the crude ethanolic extract of *C. affinis*. This method, originally detailed by VanWagenen et al. (1993), involves solvent-solvent partitioning using a series of solvents with increasing polarity (VanWagenen et al., 1993). In this study, the crude extract was sequentially fractionated with n-hexane, dichloromethane (DCM), ethyl acetate (EA), and distilled water. Each solvent targets compounds based on their polarity, with n-hexane extracting non-polar compounds, DCM extracting slightly polar compounds, EA extracting moderately polar compounds, and water-extracting highly polar compounds. After fractionation, rotary evaporation was employed to concentrate each fraction, resulting in the following yields: hexane soluble fraction (HSF, 12.7 g), DCM soluble fraction (DCMSF, 10.35 g), EA soluble fraction (EASF, 15.1 g), and water-soluble fraction (AQSF, 9.8 g). This process exemplifies the systematic and efficient isolation of different compound classes, which is characteristic of the Kupchan method.

## Phytochemical analysis

### GC-MS analysis

The whole plant extract of *C. affinis* was subjected to electron impact ionization (EI) to extract beneficial substances. This analysis has been performed utilizing a SHIMADZU GC-MS QP-2020 instrument with an AOC-20s auto-sampler and an AOC-20i auto-injector. The analysis utilised an SH Rxi 5M Sill column with dimensions of 30 m × 0.25 mm and a particle size of 0.25 μm. The carrier gas used was helium, having 1.72 mL/min flow pressure. The oven temperature adhered to a pre-established pattern, beginning at 80 °C (maintained for 2.00 min, increased at a rate of 5 °C/min) and achieving 150 °C (maintained for 5.00 min), ultimately attaining an ultimate temperature of 280 °C (maintained for 5.00 min). In splitless injection mode, a 5.0 μL injection volume was used with a split proportion of 50:1, while the injector was heated to 220 °C and the ion source to 280 °C. A mass spectrometric ionization analysis was carried out at an energy of 70 electron volts (eV), encompassing a mass range from 45 mass-to-charge ratio (m/z) to 350 m/z, during 50.0 min. A solvent cutting time of 5.0 min and a total running duration of 55.0 min were used. Bioactive substances were recognized by calculating their proportion from the entire peak area, using their retention time and MS fragment ions. The secondary metabolites were ascertained by comparing respective mass spectra with the records in the NIST08, NIST08s and NIST14 libraries. This method assisted in the establishment of structures, compound names and molecular weights of metabolites (Kim et al., 2016; Obaidullah et al., 2021).

### Phytochemical screening

The primary phytochemical analysis of crude ethanolic extract of *C. affinis* was carried out following the protocols that were established by Harborne (1998) and Edeoga et al. (2005). The existence of a wide range of secondary metabolites was investigated in this article. These metabolites included tannins, sterols, carbohydrates, quinones, alkaloids, phenolic resin, fatty oils, proteins and amino acids, glycosides, terpenoids, hormones, saponins, phlobatannins, and flavonoids (Harborne, 1998; Edeoga et al., 2005).

### Antioxidant assay

#### Total phenolic content (TPC) analysis

The Folin-Ciocalteu reagent served as the oxidizing agent, and gallic acid was used as the standard for the measurement of the total phenolic content of different fractionated extractives of *C. affinis* as per established methodology (Škerget, 2005). After adding 0.5 mL of each of the four fractionated solutions (HSF, DCMSF, EASF and AQSF) with a concentration of 2 mg/mL, 2.5 mL of a Folin-Ciocalteu reagent that had been diluted 10 times with water and 2.0 mL of a Na_2_CO_3_ solution with a concentration of 7.5% (w/v) were subsequently added. The mixtures were allowed to ferment at room temperature for 20 min. After the 20 min of incubation, an absorbance reading was taken at 760 nm using a UV spectrophotometer. The total phenols content of each sample was determined by comparing this reading to a standard curve constructed from a gallic acid solution of different concentrations. The phenolic insides of the extracts were measured in milligrams of gallic acid equivalent (mg of GAE) per gram of extract.

#### DPPH scavenging test

The antioxidant potential is generally assessed using 1,1-diphenyl-2-picrylhydrazyl (DPPH) free radical method (Brand-Williams et al., 1995). In this study, 3.0 mL of a DPPH ethanol solution with a concentration of 20 μg/mL was combined with 2.0 mL of each sample of the four fractionated extractives of *C. affinis* (HSF, DCMSF, EASF and AQSF) and control sample at various serially diluted to some concentrations (spanning from 500 μg/mL to 0.977 μg/mL). During serial dilution, the concentrations were reduced to half each of the times. Then, the mixtures were stored in a place with complete darkness at room temperature for 30 min. Following the reaction period, a UV spectrophotometer was employed to measure the absorbance at 517 nm relative to ethanol used as a blank. As a positive control, tert-butyl-1-hydroxytoluene (BHT) was employed and observed with DPPH. The succeeding equation was utilized to estimate the free radical DPPH inhibition percentage:

$$\%\;Inhibition\;of\;free\;radical\;DPPH=1-\frac{Absorbance\;of\;sample}{Absorbance\;of\;the\;control\;reaction}\times 100$$

Then % inhibitions of the test samples were plotted against the different concentrations utilized, and the IC_50_ was estimated from the graph during this phytochemical analysis.

### Preparation of biological assay

#### Test animal models

To conduct the *in vivo* experiment, 4–5 weeks of swiss-albino mice of either sex were acquired from the Animal Resource Branch of the International Centre for Diarrhoeal Diseases and Research, Bangladesh (ICDDR,B). Standard polypropylene cages with a 12-h light-dark cycle were used to house the mice. Additionally, the experiment maintained other ideal circumstances such as a controlled room temperature of 24°C ± 2 °C with a relative humidity of 60%–70%. The subjects were provided with ICDDR,B formulated rodent chow and water (*ad libitum*). During the experiments, the guidelines regarding the use and care of laboratory animals were correctly followed. Due to the high sensitivity of mice to environmental changes, they were acclimated in the experimental environment for a minimum of 3–4 days before the experiment. All the ethical rules and regulations were also implemented while designing the research and experiments. An intraperitoneal anesthesia overdose of ketamine HCl (100 mg/kg) and xylazine (7.5 mg/kg) was administered to the mice models at the end of the experiment, followed by euthanasia. The institutional ethics committee gave its stamp of approval to all experiments that involved the use of laboratory animals (Zimmermann, 1983). The “Animal Ethics Number” for the test animal models of this work is 2023–01-04/SUB/A-ERC/002 approved by the Animal Ethics Committee, State University of Bangladesh.

#### *In vivo* oral acute toxicity test

The mice were given high oral doses of 2000 mg/kg ethanol soluble *C. affinis* crude extract under normal conditions of laboratories following the “Organization for Environmental Control Development” guidelines (OECD: Guidelines 420) fixed-dose method (Rudra et al., 2020)(Van den Heuvel M., 1984). After the oral administration, several parameters were measured within 72 h. No allergic reaction, behavioral change (sedation/excitability), or lethality was found. Therefore, considering the oral acute toxicity perspective, the chosen safe doses for the antidiarrheal activity study were adjusted at 200 and 400 (mg/kg, b.w; p.o).

### *In vitro* evaluation

#### Cytotoxicity test

##### Brine shrimp lethality bioassay

The cytotoxicity of different extractives of *C. affinis* was examined on brine shrimp nauplii (*Artemia salina*), and as a reference standard anticancer substance, vincristine sulfate, was used (Meyer et al., 1982). To create a simulated saltwater solution for this experiment, 38 g of NaCl salt were dissolved in 1000 mL of distilled water. NaOH was then added to maintain a pH of 8.0. The each of the four fractionated extractives was dissolved in DMSO (dimethyl sulfoxide) at 50 µL/5 mL concentration to formulate the test model with simulated seawater. Each of the fractionated extractives and vincristine standard solution were serially diluted, starting from the concentration of 400 μg/mL to 0.78125 μg/mL and from the concentration of 20 μg/mL to 0.039 μg/mL, respectively. During serial dilution, the concentrations were reduced to half each time in both cases. Subsequently, ten fully developed live shrimps were placed in individual test tubes at room temperature (25°C ± 1 °C), and the quantity of deceased nauplii was assessed after a 24-h period.

$$Mortality\;\left(\%\right)=\frac{N1}{N0}\times 100$$

where, N0 = Number of nauplii taken; N1 = Number of nauplii death.

#### Antimicrobial assay

##### Disc diffusion test

The antimicrobial activity of four fractions of *C. affinis* ethanolic extract was assessed using the disc diffusion technique (Huys et al., 2002). Dried and sterilized filter paper discs (6 mm diameter) containing 100 µg of each test sample (HSF, DCMSF, EASF, AQSF) were placed on nutrient agar medium previously inoculated with test bacteria and fungi strains. Commercial antibiotic discs of Azithromycin, Amoxicillin, and Ciprofloxacin (30 µg/disc) and an antifungal disc of Fluconazole (30 µg/disc) were used as positive controls, while blank discs served as negative controls. To ensure even distribution, the plates were inverted and kept at 4°C for 24 h, then incubated at 37°C for another 24 h. Zones of inhibition, indicating antibacterial activity, were measured in millimeters (Alam et al., 2021a). Antibacterial activity was evaluated on the clinically isolated strains of gram-positive bacteria (*S. lutea, B. megaterium, S. aureus*, *B. subtilis*, *B. cereus*), gram-negative bacteria (*V. mimicus*, *P. aeruginosa, S. typhi, S. paratyphi, E. coli*, *S. dysenteriae,* and *V. parahemolyticus*) and fungi strains (*A. niger*, *Sacharomyces cerevacae* and *C. albicans*)

### *In vivo* evaluation

#### Antidiarrheal assay

##### Castor oil-induced diarrhea test

Following the protocol outlined by previous research, the anti-diarrheal activity of different fractions of *C. affinis* was tested on castor oil-induced diarrheal mice (Shoba and Thomas, 2001; Rudra et al., 2020). Each mouse was administered 1 mL of highly pure analytical grade castor oil to induce diarrhea, and the total number of feces excreted was recorded. Mice were divided into four groups: control, positive control, and two test groups, each containing five mice. The control group received 10 mL/kg of 1% Tween 80 in water orally, while the positive control group received 5 mg/kg of loperamide orally. The test groups were given *C. affinis* extract fractions at 200 mg/kg and 400 mg/kg, respectively. After treatment, diarrhea was induced with castor oil, and each mouse was housed in a separate cage with hourly floor lining changes. The anti-diarrheal effects were assessed by comparing the test groups to the control group, with fecal stool counts recorded over a 4-h observation period. The percent inhibition of diarrhea was calculated using the following equation.

$$\%\;inhibition\;of\;defecation=\frac{Mean\;number\;of\;defecation\;by\;control-Mean\;number\;of\;defecation\;by\;test\;samples\;or\;standard}{Mean\;number\;of\;defecation\;by\;control}\times 100$$

#### Analgesic assay

##### Acetic acid-induced writhing test

The pain-relieving effectiveness of various fractions from *C. affinis* was examined using the acetic acid-induced writhing test methodology (Ahmad et al., 2010). The procedure is the injection of acetic acid into mice, resulting in a distinct writhing reaction caused by pain. The decrease in the number of writhes serves as an indicator of the analgesic agent’s efficacy. Groups of five mice each were designated as control, positive control, and test groups for four different fractions. All mice received 0.1 mL of acetic acid intraperitoneally to induce writhing. The positive control group was then orally given 5 mg/kg of diclofenac sodium. The test groups received *C. affinis* extract fractions at doses of 200 mg/kg and 400 mg/kg orally. Five minutes after acetic acid injection, the number of writhing movements was recorded over 25 min. The percentage of writhing inhibition was calculated using the following equation.

$$\%\;Inhibition\;of\;writhing=\frac{Control\;writhing\;response-Test\;writhing\;response}{Control\;writhing\;response}\times 100$$

### Statistical analysis

The statistical analysis was performed using GraphPad Prism 5.2 (GraphPad Software, Inc., La Jolla, CA, United States), and the results were expressed as mean ± standard error (SEM). One-way variance analysis (ANOVA) and Dunnett’s test were used to determine statistical significance; *p < 0.5, **p < 0.01, and ***p < 0.001 were deemed statistically significant.

### Molecular docking and ADME/T studies

#### Docking software

The metabolites identified from different extracts of the whole plant extract of *C. affinis* underwent computational docking study utilizing well-known software tools, including PyRx, PyMoL 2.3, Discovery Studio 4.5, and Swiss PDB viewer (Hasnat et al., 2023).

#### Ligand preparation

The phytochemicals listed in Table 1 were queried in the PubChem database (https://pubchem.ncbi.nlm.nih.gov/) and their 3D structures were downloaded in SDF format. Additionally, the 3D SDF structures of loperamide (PubChem CID- 3955), amoxicillin (PubChem CID- 33613), and ascorbic acid (PubChem CID- 54670067) were obtained as standard references. These ligands, alongside their particular PubChem CIDs, were successively stacked into Discovery Studio 4.5. Strikingly, the Pm6 semi-empirical method was utilized to optimize all phytochemicals, improving docking precision (Taher et al., 2023).

**TABLE 1:** GC-MS analysis of different fractions of whole plant of *Colocasia affinis* Schott.

ID	Name	R.Time	m/z	Peak area	Conc. (%)	Structure
Dichloromethane Fraction						
1	Pentanoic acid, 4-nitro-, methyl ester	5.797	55	22,768	1.1	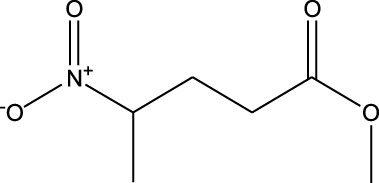
2	Azulene	8.482	128	55,119	2.664	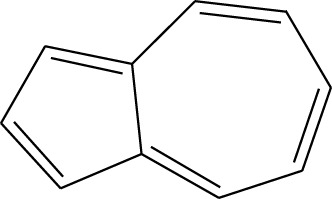
3	9-Methoxybicyclo[6.1.0]nona-2,4,6-triene	12.81	115	67,215	3.249	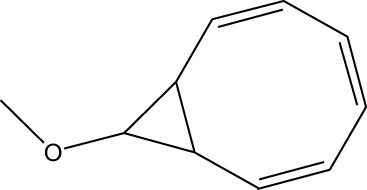
4	Benzenemethanol, .alpha.-methyl-.alpha.-propyl-	14.685	115	38,539	1.863	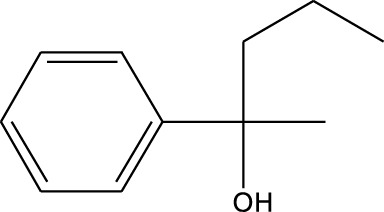
5	3-Tetradecene, (Z)-	14.806	55	50,851	2.458	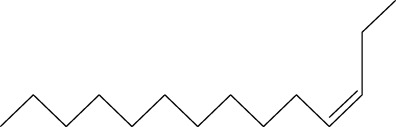
6	Estragole	14.979	115	117,376	5.673	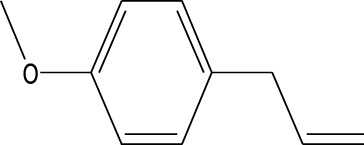
7	Dodecane, 2,6,11-trimethyl-	17.795	57	35,769	1.729	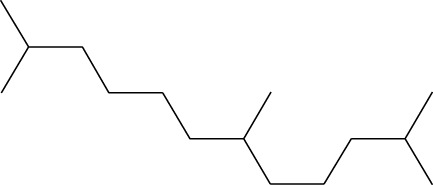
8	Phenol, 2,6-bis(1,1-dimethylethyl)-	18.093	57	53,997	2.61	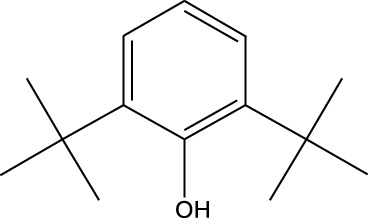
9	Phenol, 3,5-bis(1,1-dimethylethyl)-	18.42	191	1,012,864	48.952	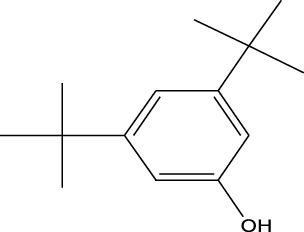
10	1-Undecene	20.335	55	28,753	1.39	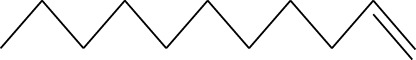
11	3-Hexadecene, (Z)-	21.002	55	12,865	0.622	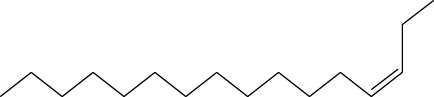
12	Pentafluoropropionic acid, decyl ester	26.09	55	20,901	1.01	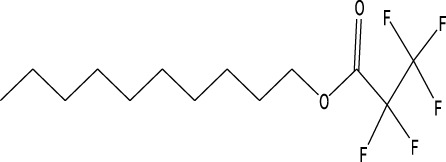
13	Undecanoic acid, 10-methyl-, methyl ester	30.906	74	90,335	4.366	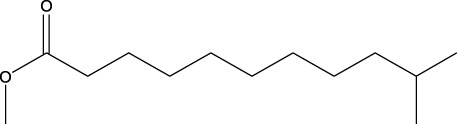
14	9,12-Octadecadienoic acid, methyl ester, (E,E)-	36.062	67	41,423	2.002	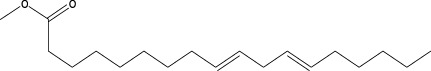
15	9-Octadecenoic acid (Z)-, methyl ester	36.272	55	168,852	8.161	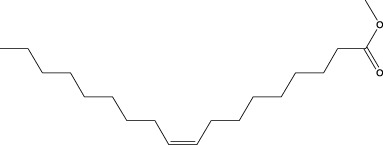
16	13-Docosenoic acid, methyl ester, (Z)-	41.483	55	34,688	1.676	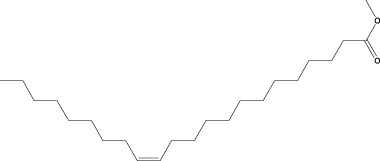
17	15-Tetracosenoic acid, methyl ester, (Z)-	45.993	55	99,438	4.806	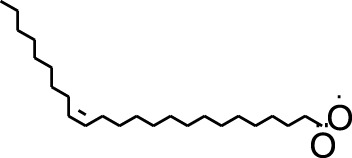
18	Di-n-octyl phthalate	46.518	149	63,298	3.059	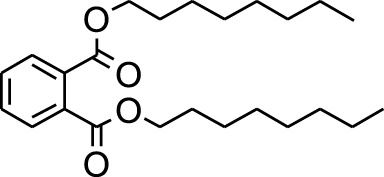
19	4,6-Bis(1,1-dimethylethyl)-1,1′-biphenyl-2-ol	49.21	57	35,463	1.714	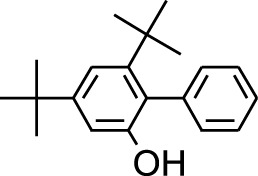
20	Cyclotetrasiloxane, octamethyl-	58.96	281	18,567	0.897	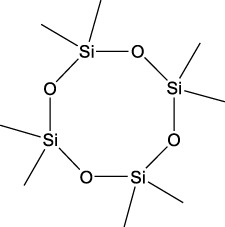
Ethyl Acetate Fraction						
1	Estragole	14.977	115	79,912	8.399	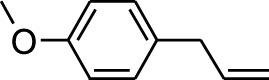
2	Heptadecane, 2,6,10,15-tetramethyl-	17.789	57	43,339	4.555	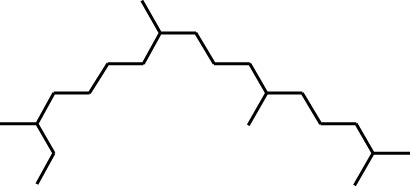
3	Phenol, 3,5-bis(1,1-dimethylethyl)-	18.203	191	29,644	3.116	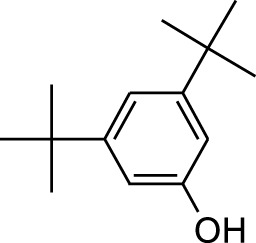
4	Phenol, 2,6-bis(1,1-dimethylethyl)-	18.414	191	608,040	63.907	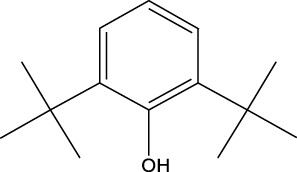
5	Tetracosane	19.195	57	53,483	5.621	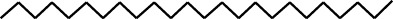
6	Diisooctyl phthalate	46.511	149	137,034	14.403	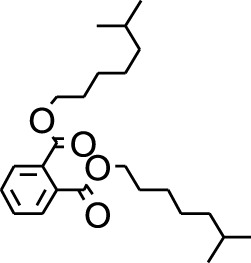
n-Hexane Fraction						
1	9-Oxabicyclo[6.1.0]nonan-4-ol	3.89	55	14,056	1.868	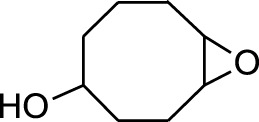
2	Cyclohexane, 1,1-dimethoxy-	4.137	101	18,311	2.433	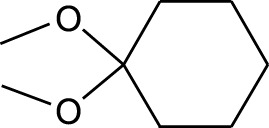
3	Epoxy-linalooloxide	5.752	83	25,016	3.325	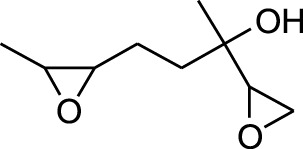
4	n-Tridecan-1-ol	8.553	55	15,768	2.096	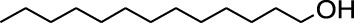
5	9-Methoxybicyclo[6.1.0]nona-2,4,6-triene	12.805	115	24,483	3.254	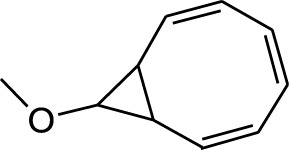
6	1-Tetradecene	14.79	55	23,527	3.127	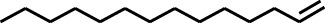
7	Phenol, 3,5-bis(1,1-dimethylethyl)-	18.403	191	631,291	83.898	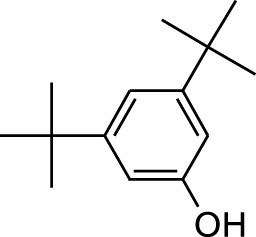
Aqueous Fraction						
1	Undecane, 5,7-dimethyl-	4.98	57	356,893	6.911	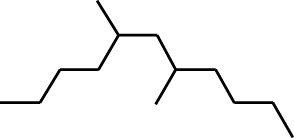
2	Decane, 2,3,5,8-tetramethyl-	5.986	57	146,470	2.836	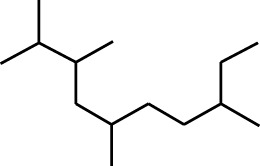
3	Dodecane	8.783	57	179,041	3.467	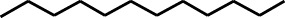
4	Tridecane	9.06	57	156,139	3.023	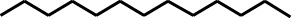
5	Octane, 2,3,7-trimethyl-	9.156	57	134,094	2.597	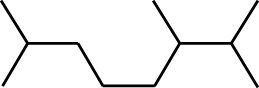
6	Pentadecane	10.167	57	70,521	1.366	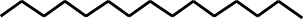
7	Undecane, 2,4-dimethyl-	10.652	57	174,438	3.378	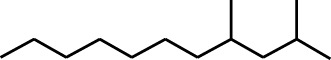
8	Hexane, 3,3-dimethyl-	10.945	71	96,921	1.877	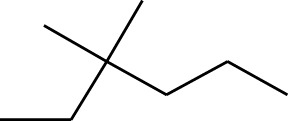
9	Dodecane, 2,6,10-trimethyl-	11.045	57	341,741	6.617	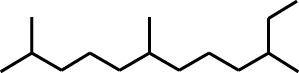
10	Dodecane, 4-methyl-	11.73	71	59,121	1.145	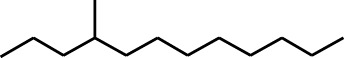
11	1-Octanol, 2-butyl-	11.859	57	120,287	2.329	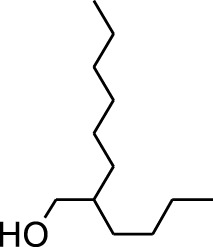
12	Decane, 4-ethyl-	12.06	57	83,639	1.62	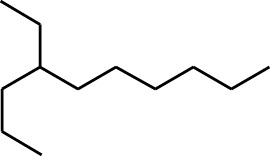
13	Decane, 2,3,5,8-tetramethyl-	12.513	57	270,411	5.236	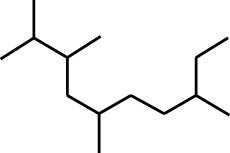
14	9-Methoxybicyclo[6.1.0]nona-2,4,6-triene	12.765	115	47,123	0.912	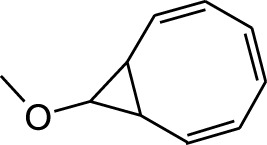
15	Nonadecane	12.82	57	45,357	0.878	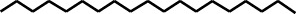
16	Heneicosane	13.877	57	97,434	1.887	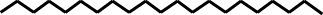
17	Heptadecane, 2,6,10,15-tetramethyl-	14.097	57	93,615	1.813	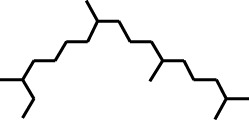
18	Estragole	14.942	115	48,227	0.934	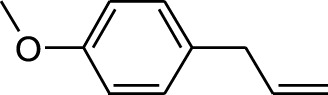
19	Tridecane	15.036	57	252,338	4.886	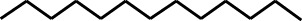
20	Pentadecane	15.374	57	119,779	2.319	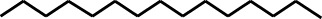
21	Octadecane	15.512	57	143,147	2.772	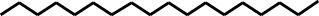
22	Tetradecane, 4-methyl-	15.69	71	64,699	1.253	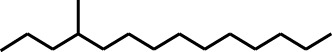
23	Hexadecane	16.159	57	49,023	0.949	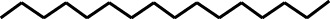
24	Heneicosane	16.787	57	167,179	3.237	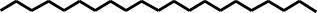
25	Tetradecane, 4,11-dimethyl-	16.932	71	141,695	2.744	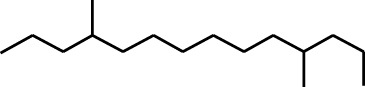
26	Tetracosane	16.992	57	193,021	3.738	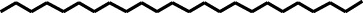
27	Heptadecane, 2,6,10,15-tetramethyl-	17.644	57	143,375	2.776	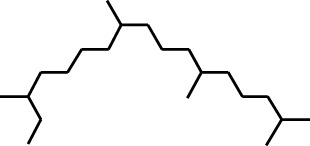
28	2,3-Dimethyldodecane	17.765	57	349,190	6.761	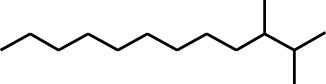
29	Pentadecane, 2,6,10-trimethyl-	18.007	57	105,481	2.042	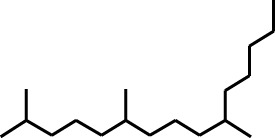
30	Phenol, 3,5-bis(1,1-dimethylethyl)-	18.385	191	554,943	10.746	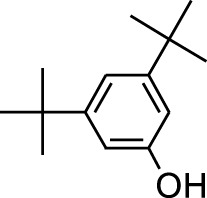
31	Hexadecane	19.026	57	95,003	1.84	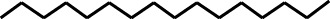
32	Heptadecane, 2,6,10,15-tetramethyl-	19.175	57	171,449	3.32	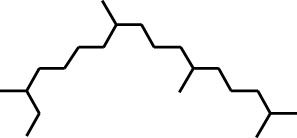
33	Nonadecane	19.483	57	29,407	0.569	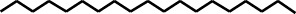
34	Eicosane	24.043	57	63,206	1.224	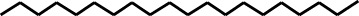

#### Receptor preparation

A computational docking analysis was performed to find the antidiarrheal, antimicrobial, and anti-inflammatory potentiality of 48 identified metabolites from *C. affinis* in the whole plant. The 3D crystalized structures of target receptors utilized in this study were sourced from the Protein Data Bank RCSBPDB (https://www.rcsb.org) (Prlić et al., 2010) These structures include the kappa-opioid receptor (KOR) [PDB: 6VI4] (Che et al., 2020) and human delta-opioid receptor (DOR) [PDB: 4RWD] (Fenalti et al., 2015) for antidiarrheal docking research. Additionally, the Beta-ketoaryl-ACP synthase 3 receptor (KAS) [PDB: 1HNJ] (Qiu et al., 2001) and Dihydrofolate reductase (DHFR) [PDB: 4M6J] (Khatun et al., 2021) were used for antimicrobial docking analysis. In contrast, Glutathione reductase (GLR) [PDB: 3GRS] (Qiu et al., 2001) and Urate oxidase (URO) [PDB: 1R4U] (Retailleau et al., 2004) were employed for antioxidant docking investigations. Again, Epidermal growth factor receptor (EGFR) [PDB: 1XKK] (El Azab et al., 2021) and B-cell lymphoma 2 (BCL-2) [PDB:4LXD] (Nurdiansyah et al., 2023) were picked for investigation of cytotoxic activity. Last but not least, in the case of analgesic activity, Cyclooxygenase- 2 (COX-2) [PDB ID: 1CX2] (Muhammad et al., 2015) and Tumor necrosis factor alpha (TNF-α) [PDB ID: 2AZ5] (Kumar et al., 2021) were selected for site-specific docking. These proteins or receptors were stored in the PDB format. These proteins were processed using PyMOL 2.3 to remove water molecules and ligands/residues. Subsequently, non-polar hydrogen atoms were added to the biomolecules and optimized to their lowest energy state using the energy minimization tool in the Swiss PDB viewer (Hasnat et al., 2023).

#### Ligand-receptor bindings

Computer-based ligand-protein interaction analysis was conducted to predict probable binding interactions between phytochemicals and their target proteins. This process involved employing the advanced PyRxAutodock Vina for molecular docking and utilizing semiflexible modeling techniques. Initially, the protein was laden and organized as the intended macromolecule. Amino acids derived from literature sources and their matching identification codes were meticulously chosen to guarantee the accurate attachment of ligands only to the intended macromolecule.

In the case of the KOR [PDB: 6VI4], specific amino acids from the A chain, including Leu 103, Leu 107, Ser 136, Ile 137, Try 140, Ile 180, Trp 183, Leu 184, Ser 187, Ile 191, Leu 192, Ile 194, and Val 195, were chosen for docking (Shompa et al., 2024). Ala9, Ile16, Lys54, Lys55, Thr56, Leu75, Ser76, Arg77, Glu78, Arg91, Ser92, Leu93, Gly117, Ser118, Ser119, and Val120 were picked for DHFR [PDB: 4M6J] (Khatun et al., 2021) However, the site specific amino acids for EGFR [PDB: 1XKK] were Leu 718, Val 726, Ala 743, Lys 745, Met 766, Lys 775, Arg 776, Leu 777, Leu 788, Thr 790, Gln 791, Leu 792, Met 793, Gly 796, Cys 797, Leu 799, Asp 800, Arg 803, Leu 844, Thr 854, Asp 855, and Phe 856 (Taher et al., 2023). Also, in the case of COX-2 [PDB ID: 1CX2] amino acids, including His 90, Gln 192, Val 349, Leu 352, Ser 353, Tyr 355, Tyr 385, Ala 516, Phe 518, Val 523, Ala 527, and Ser 530 were selected for docking. Furthermore, Tyr59, Tyr119, Leu 120, Gly 121, and Tyr 151 amino acids within the A chain, along with Tyr 59, Ser 60, Gln 61, Tyr 119, Leu 120, and Gly 121 amino acids in the B chain of TNF- α, were selected for site-specific docking, as detailed by (Shahriar et al., 2024).

Prankweb was utilized to determine the probable active site of the rest of the target proteins (Jendele et al., 2019). Val 62, Leu 65, Gly 66, Leu 69, Val 70, Phe 72, Gly 73, Tyr 77, Pro 315, Val 316, Ala 319, Phe 325, Cys 328, Phe 329, Gln 331, and Leu 332 of were picked for A chain of DOR [PDB: 4RWD]. Consequently, prankweb was used to select amino acids including Trp 32, Arg 36, Thr 37, Thr 81, Ala 109, Ala 110, Ala 111, Cys 112, Leu 142, Gly 152, Ile 155, Ile 156, Phe 157, Leu 189, Thr 190, Leu 191, Leu 205, Met 207, Gly 209, Asn 210, Val 212, Phe 213, Ala 216, Leu 220, His 244, Ala 246, Asn 247, Ile 250, Asn 274, Glu 302, Ala 303, Phe 304, Gly 306, and Gly307 for KAS [PDB: 1HNJ], as well as Ile 26, Gly 27, Gly 29, Ser 30, Gly 31, Val 49, Glu 50, Ser 51, Lys 52, Gly 56, Thr 57, Cys 58, Val 61, Gly 62, Cys 63, Lys 66, Lys 67, Gly 128, His 129, Ala 130, Ala 155, Thr 156, Gly 157, Gly 158, Met 159, Ser 177, Phe 181, Tyr 197, Ile 198, Glu 201, Met 202, Arg 291, Asn 294, Leu 298, Asp 331, Leu 337, Leu 338, Thr 339, Pro 340, Ala 342, Val 370, and Phe 372 for GLR [PDB: 3GRS]. Additionally, amino acids Phe 159, Phe 162, Thr 168, Leu 170, Lys 171, Thr 173, Arg 176, Ile 177, Val 227, Gln 228, Asn 254, His 256, Tyr 257, Phe 258, Glu 259, Phe 278, Pro 284, Gly 286, Leu 287, Ile 288 for URO [PDB: 1R4U] (Jendele et al., 2019) were selected for site-specific docking. Lastly, Phe 101, Asp 108, Phe 109, Met 112, Val 130, Glut 133, Leu 134, Arg 143, Ala 146, Glu 149, Phe 150, and Val 153 were selected for BCL-2 [PDB: 4LXD].

As for the standard, loperamide was used against KOR and DOR, while amoxicillin was chosen for KAS and DHFR. Ascorbic acid was utilized against GLR and URO, while lapatinib was utilized as the standard in the case of EGFR and BCL-2. Finally, diclofenac was employed for COX-2 and TNF-α. Furthermore, the ligands’ PDB files were uploaded and converted to pdbqt format using the Open Babel feature in PyRxAutoDock Vina software. This allowed us to find the best binding interactions while docking with the chosen macromolecules.

The grid box was generated by ensuring that the active binding sites of the protein were enclosed within the designated box, as determined through grid mapping. The KOR grid box is positioned with a center at X = 41.2113828749, Y = −54.1291662632, and Z = −22.5997323179, with dimensions X = 16.7196551518, Y = 27.2876737202, and Z = 16.5467118974. For DOR, the center was at X = −57.2878363954, Y = 2.11011895459, and Z = 53.1963612028, with dimensions x = 22.8756741846, y = 19.0839303805, and z = 20.669369331. KAS maintained a center at X = 28.9339098379, Y = 17.5191106637, Z = 31.6525120583, with dimensions X = 26.0697735011, Y = 34.8553116374, and Z = 22.3970043137. DHFR had a center at X = 2.99808311921, Y = −3.54674634211, and Z = −18.5871843936, with dimensions X = 19.9283113118, Y = 27.7380574575, and Z = 27.1400591135. GLR was positioned at X = 60.8957977373, Y = 50.7392192752, Z = 15.8962732414, with dimensions X = 35.4645585801, Y = 24.3261287651, and Z = 26.4858683614. Again, URO was fixed at a center of X = 31.325851641, Y = 25.0311624292, and Z = 44.6854822753, with dimensions X = 19.2797025429, Y = 27.2857277055, and Z = 27.0044877641. For EGFR, the grid box peaked at a center of X = 15.9440369259, Y = 34.4198880619, and Z = 35.8044407692, with dimensions X = 24.7750487506, Y = 19.7793076754, and Z = 32.2557047924. Conversely, BCL-2 was centered at X = 27.0730446356, Y = 28.4708961863, and Z = 5.29432825771, with dimensions X = 16.1526029895, Y = 20.5518919333, and Z = 22.8016796153. Also, center X = 23.011633329, Y = 20.9981639946, and Z = 15.518303992 and dimension X = 21.4798597234, Y = 18.5617032509, and Z = 23.8823675559 were maintained for COX-2. For TNF-α, the center was positioned at X = −19.7381611157, Y = 74.2118779135, and Z = 37.7182960594, with dimensions X = 19.5448302136, Y = 22.3686683962, and Z = 15.1249201064.

The leftover settings were set to their default configurations during the docking process. After that, AutoDock Vina (version 1.1.2) guaranteed a uniform set of circumstances for computer-dependent molecular docking of the phytochemicals. In the end, BIOVIA Discovery Studio version 4.5 was used to carefully examine all docking tests, helping to identify the best models through a thorough analysis of both 2D and 3D configurations.

#### ADME/T analysis

In contemporary drug design, computational methods focusing on pharmacokinetics (absorption, distribution, metabolism, excretion, and toxicology) and assessing bioavailability through drug-like properties have gained substantial traction. In drug discovery, ADMET analyses are pivotal tools for unraveling the pharmacological landscape (http://biosig.unimelb.edu.au/pkcsm/prediction). Furthermore, Swiss ADME (http://www.sib.swiss), an online platform, was employed to forecast drug likeliness based on Lipinski rules and pharmacokinetic parameters for various compounds. According to Lipinski’s criteria, a compound is considered orally viable if it satisfies the following conditions: the compound should have a molecular weight of less than 500 atomic mass units (amu), no more than 5 hydrogen bond donor sites, no more than 10 hydrogen bond acceptor sites, and a lipophilicity value (LogP) of 5 or less (Taher et al., 2024).

## Results

### Phytochemical analysis

#### GC-MS analysis

During the gas chromatography-mass spectrometry (GC-MS) analysis, various portions of the plant being examined exhibited a combined total of 67 peaks, each representing a bioactive compound. The identity of these molecules was determined by comparing their molecular mass, chemical formula, and peak retention time with the properties of compounds recognized in the NIST library. This investigation seeks to provide insight into the various bioactive metabolitess found in several extracts of *C. affinis*, which make a major contribution to the existing body of knowledge.

**FIGURE 2:**
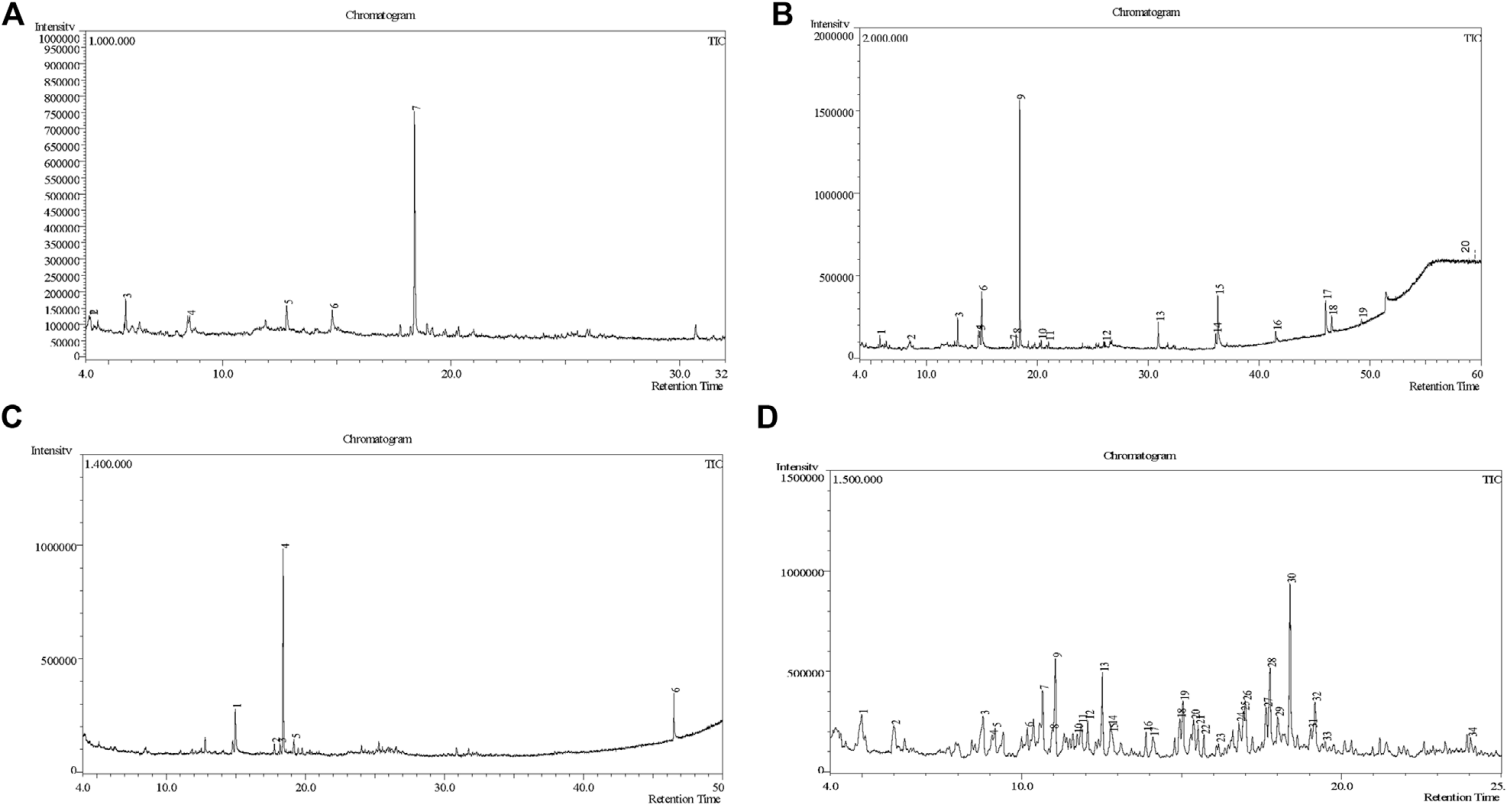
GC-MS chromatogram of different fractions of ethanolic extract of *Colocasia affinis* whole plant, here **(A, B,C)** and **(D)** represent n-hexane, DCM, ethyl acetate and aqueous fractions, respectively.

A DCM-based extract of *C. affinis* revealed the presence of 20 chemicals, as detailed in Table 1 and depicted in Figure 2. The relative concentration of each compound was expressed as a peak area percentage. Noteworthy compounds with substantial prevalence included Phenol, 3,5-bis(1,1-dimethylethyl)- (48.952%), 9-Octadecenoic acid (Z)-, methyl ester (8.161%), Estragole (5.673%), 15-Tetracosenoic acid, methyl ester, (Z)- (4.806%), Undecanoic acid, 10-methyl-, methyl ester (4.366%), 9-Methoxybicyclo[6.1.0]nona-2,4,6-triene (3.249%), and Di-n-octyl phthalate (3.059%). The rest of the compounds are found within a concentration of less than 3%. The retention time, mass/charge ratio and the peak area are also predicted in Table 1.

Six compounds were identified in the case of ethyl acetate extract of the plant (Table 1, Figure 2). Here Phenol, 2,6-bis (1,1-dimethyl ethyl)- presented with highest 63.907% concentration, followed by Diisooctyl phthalate with 14.403%, Estragole with 8.399%, Tetracosane with 5.621%, Heptadecane, 2,6,10,15-tetramethyl- with 4.555%, and Phenol, 3,5-bis (1,1-dimethyl ethyl)- with 3.116% concentration.

Seven peaks were detected in the plant hexane extract. The compound with the highest concentration is Phenol, 3,5-bis(1,1-dimethylethyl), constituting 83.898% of the total. Additionally, Epoxy-linalool oxide, 9-methoxybicyclo [6.1.0] nona-2,4,6-triene, and 1-tetradecene are present at concentrations of 3.325%, 3.254%, and 3.127%, respectively.

The highest 34 compounds have been identified from the aqueous fraction of the *C. affinis.* The most prominent peak area with a concentration of 10.746% was observed for Phenol, 3,5-bis(1,1-dimethylethyl)-. Other notable compounds included Undecane, 5,7-dimethyl- (6.911%), 2,3-Dimethyldodecane (6.761%), Dodecane, 2,6,10-trimethyl- (6.617%), Decane, 2,3,5,8-tetramethyl- (5.236%), Tridecane (4.886%), Tetracosane (3.738%), Dodecane (3.467%), Undecane, 2,4-dimethyl- (3.378%), Heptadecane, 2,6,10,15-tetramethyl- (3.320%), and Heneicosane (3.237%). Details are outlined in both Table 1 and Figure 2.

#### Phytochemical screening

Several extracts of *C. affinis* were subjected to preliminary phytochemical analysis, which identified tannins, sterols, carbohydrates, quinones, saponins, alkaloids, phenolic compounds, flavonoids, glycosides, terpenoids, and steroids (Table 2).

**TABLE 2:** Preliminary phytochemicals screening of different fractions of *Colocasia affinis*.

Phytochemicals	HSF	DCMSF	EASF	AQSF
Tannins	+	+	+	+
Sterols	+	+	-	-
Carbohydrates	+	+	-	-
Quinone	-	-	+	-
Saponins	+	-	+	+
Alkaloids	-	-	+	+
Phenolic compound	+	+	+	+
Phlobatannins	-	-	-	-
Fixed oils and fats	-	-	-	-
Flavonoids	+	+	+	+
Protein and amino acid	-	-	-	-
Glycosides	+	+	+	+
Terpenoids	+	+	+	+
Steroids	+	+	+	+

(+) = present, (−) = absent.

#### Antioxidant assay

##### Total phenolic content of different *C. affinis* extractives

**FIGURE 3:**
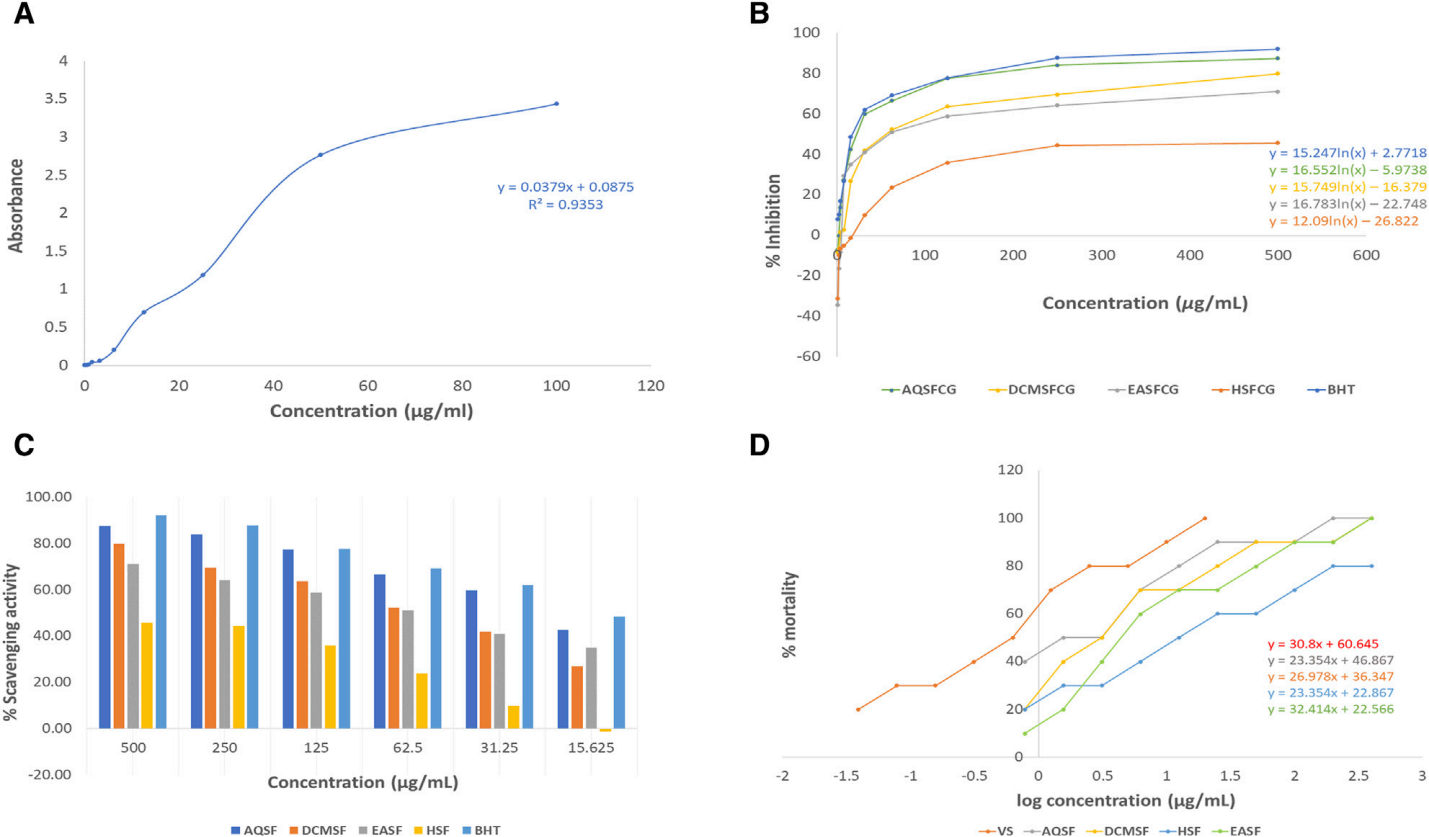
Analysis of Cytotoxic and Antioxidant Attributes of *Colocasia affinis* Fractions: **(A)** Standard curve of gallic acid for determining total phenolic content in various fractions. **(B)** Linear regression equations (IC_50_) for butylated hydroxytoluene (BHT) and different fractions observed with DPPH assay. **(C)** Percentage of radical scavenging activities of BHT and different fractions. **(D)** Linear regression equations (LC_50_) for vincristine sulfate (VS) and different fractions.

The extent of total phenolic content of different fractions of *C. affinis* varied from 7.98 to 57.23 mg of GAE/gm of extractives (Table 3). The plant’s aqueous fraction (AQSF) had the highest concentration of phenolic content, whereas the dichloromethane soluble fraction (DCMSF) displayed a significant proportion. Figure 3 shows the standard curve of gallic acid used to determine the total phenolic content of various extracts.

**TABLE 3:** Total phenolic content, free radical scavenging and cytotoxic activities of different extracts of *C. affinis* with respective standards.

Test sample	Antioxidant		Cytotoxic
	Total phenolic content (mg of GAE/gm of extract)	IC_50_ value	LC_50_ value
HSF	7.98	574.23	14.51
DCMSF	44.46	67.63	3.21
EASF	28.36	76.2	7.02
AQSF	57.23	29.4	1.36
BHT/VS	-	22.13	0.451

##### Effect of different *C. affinis* extractives on DPPH free radical scavenging property in phytochemical analysis

The DPPH free radical scavenging investigation showed that several fractionated extracts of *C. affinis* exhibited promising free radical scavenging properties in phytochemical analysis. AQSF manifested the most pronounced scavenging attributes, with an IC_50_ value of 29.40 μg/mL, compared to the standard BHT, with an IC_50_ value of 22.13 μg/mL. The IC_50_ values, calculated by linear regression equation, and the respective percent (%) scavenging attribute of BHT and the fractions are summarized in Table 3 and Figure 3.

#### In vitro evaluation

##### Effect of different *C. affinis* extractives on brine shrimp lethality bioassay

The LC_50_ values of different fractions of *C. affinis* and the standard were 14.51 μg/mL (HSF), 7.02 μg/mL (EASF), 3.21 μg/mL (DCMSF), 1.36 μg/mL (AQSF), and 0.451 μg/mL (VS), respectively. The respective data are presented in Figure 3 and Table 3. Among all the fractions, AQSF showed the most promising cytotoxicity activity.

##### Effect of different *C. affinis* extractives on disc diffusion assay

The antibacterial activity of all the partitions was tested against seven strains of gram-positive bacteria, seven strains of gram-negative bacteria, and three strains of fungi. As a reference, standard Azithromycin, Amoxicillin, Ciprofloxacin, and Fluconazole were taken to test the respective antimicrobial activity. The zone of inhibition (ZOI) of the test samples ranged from 5 mm to 20 mm, summarized in Table 4. The AQSF and DCMSF showed considerable antibacterial activity, whereas the EASF and AQSF exhibited promising antifungal attributes. As per ZOI, the fractionated extracts exerted notable antimicrobial activities against *B. cereus*, *B. megaterium*, *S. aureus*, *S. lutea*, *E. coli*, *P. aeruginosa*, *S. paratyphi, S. dysenteriae, A. niger* and relatively lower ZOI against *B. subtilis*, *S. typhi, V. mimicus*, *V. parahemolyticus, C. albicans* and *Sacharomyces cerevacae*.

**TABLE 4:** The antimicrobial activity of *Colocasia affinis* extracts and standard against Gram-positive bacterial, Gram-negative bacterial and fungal strains.

Test Microorganisms	Zone of inhibition (mm)						
	Azithromycin (30 μg/disc)	Amoxicillin (30 μg/disc)	Ciprofloxacin (30 μg/disc)	AQF (100 μg/disc)	DCMSF (100 μg/disc)	EASF (100 μg/disc)	HSF (100 μg/disc)
Gram-Positive Bacteria							
*Bacillus cereus*	37	35	31	15	12	10	8
*Bacillus megaterium*	35	32	30	14	11	9	5
*Bacillus subtilis*	34	28	32	9	10	9	6
*Staphylococcus aureus*	41	39	33	18	17	14	9
*Sarcina lutea*	37	35	29	16	13	15	8
Gram-Negative Bacteria							
*Escherichia coli*	38	36	34	17	14	11	12
*Pseudomonas aeruginosa*	41	37	38	20	13	10	7
*Salmonella paratyphi*	30	31	27	15	15	7	-
*Salmonella typhi*	39	31	36	18	16	8	-
*Shigella dysenteriae*	37	32	33	17	15	12	9
*Vibrio mimicus*	31	28	26	12	15	-	-
*Vibrio* *parahemolyticus*	40	35	33	13	16	12	11
Fungi							

Test Microorganisms	Fluconazole (30 μg/disc)			AQF (100 μg/disc)	DCMSF (100 μg/disc)	EASF (100 μg/disc)	HSF (100 μg/disc)
*Aspergillus niger*	45			12	5	9	-
*Candida albicans*	38			-	-	-	-
*Sacharomyces* *cerevacae*	41			9	-	7	-

#### In vivo evaluation

##### Effect of different *C. affinis* extractives on castor oil-induced diarrhea

DCMSF, AQSF, HSF, and EASF fractions at 200 and 400 mg/kg doses exhibited significant (*p* < 0.05, *p* < 0.01, *p* < 0.001) reduction in the number of feces (Table 5). In terms of wet feces number, DCMSF, AQSF, HSF and EASF demonstrated percentages of diarrhea inhibition respectively by 39.53%, 18.6%, 11.63% and 6.98% at 200 mg/kg dose; while at 400 mg/kg dose the values were 51.16%, 27.91%, 20.93% and 16.28% respectively. Whereas, the value of the standard loperamide was 67.44%.

**TABLE 5:** The antidiarrheal and analgesic effect of different extracts of *Colocasia affinis* respectively on castor oil-induced and acetic acid-induced test in mice.

Animal group with respective doses (ml/kg or mg/kg, b.w; p.o)	Number of diarrheal feces (mean ± SEM)	% Reduction of diarrhea	Number of writhing (mean ± SEM)	% Reduction of writhing
CTL	8.6 ± 0.64	-	17.2 ± 0.38	-
STD (Loperamide/Diclofenac sodium)	2.8 ± 0.56***	67.44	3.8 ± 0.2***	77.91
DCMSF 200	5.2 ± 0.16***	39.53	10.4 ± 0.51***	39.53
DCMSF 400	4.2 ± 0.56*	51.16	8.2 ± 0.37***	52.33
AQSF 200	7 ± 0.8***	18.6	10.8 ± 0.58***	37.21
AQSF 400	6.2 ± 0.56***	27.91	10.2 ± 0.8**	40.70
HSF 200	7.6 ± 0.24***	11.63	14 ± 0.32***	18.60
HSF 400	6.8 ± 0.56***	20.93	13 ± 0.45***	24.42
EASF 200	8 ± 0.8***	6.98	14.4 ± 0.51***	16.28
EASF 400	7.2 ± 0.56***	16.28	12 ± 0.55***	30.23

Values are expressed as Mean ± SEM (n = 5); CTL, negative control; STD, positive control; ***p< 0.001, **p< 0.01, *p< 0.05 compared to control compared to negative control.

##### Effect of different *C. affinis* extractives on acetic acid-induced writhing in mice model

DCMSF, AQSF, HSF, and EASF fractions at 200 and 400 mg/kg doses exhibited significant (*p* < 0.05, *p* < 0.01, *p* < 0.001) analgesia with a considerable percent reduction of acetic acid-induced writhing compared to the standard diclofenac sodium (Table 5). Though the sample extracts did not exhibit dose-dependent percent reduction except the EASF, the DCMSF showed a 52.33% reduction compared to the standard of 77.91%.

### Molecular docking and ADME/T analysis

The whole plant extract of *C. affinis* revealed the presence of 48 identified compounds, which underwent computational docking tests against various receptors. Table 6 displays the binding strengths of multiple substances. Notably, for target KOR, compound C19 displayed a significant binding affection with a value of −6.9 kcal/mol, followed by C9 (−5.8 kcal/mol). C8 and C14 exhibited the same binding affinity (−5.7 kcal/mol), comparable to the standard Loperamide with a value of −7.7 kcal/mol. Regarding receptor DOR, C2, C4, C8, and C37 demonstrated the most significant binding affinity with a value of −7 kcal/mol. However, the standard Loperamide outperformed them with a binding strength of −8.9 kcal/mol. Moreover, C19 demonstrated the highest binding affinity against KAS, scoring −7.1 kcal/mol, while Compound 23 exhibited promising affinity with a value of −6.4 kcal/mol. C12 and C21 also displayed strong affinities for binding to the receptor, with values of −6.3 kcal/mol. The standard Amoxicillin had a binding affinity of −7.1 kcal/mol. Compared to the standard Amoxicillin binding score of −7.6 kcal/mol, C19 demonstrates a significant affinity for DHFR with a binding score of −7.4 kcal/mol. Additionally, C8, C9, C18, and C23 also exhibit noteworthy binding affinity to the receptor, with scores of −6.1, −6.4, −6.1, and −6.3 kcal/mol, respectively Moving to receptor GLR, C23 exhibited the highest binding affinity at −7.3 kcal/mol, and C19 also showed strong binding affinity with a score of −7 kcal/mol. Additionally, C12 and C18 displayed strong affinities for binding to 3GRS, with values of −6.2 and −6.9 kcal/mol, respectively. On the other hand, standard Ascorbic acid possessed a binding ability of −6.4 kcal/mol. Furthermore, C18 scored −5.4 kcal/mol against 3GRS, while C8 and C9 demonstrated the same prominent affinities of −5.7 kcal/mol. Interestingly, C19 exhibited very notable activity towards URO with a value of −6.8 kcal/mol, surpassing the standard Ascorbic acid, which scored −5.3 kcal/mol. Regarding EGFR, C19 exhibited the lowest affinity score at −7.8 kcal/mol, followed by C23 with a score of −7.4 kcal/mol. Additionally, C12 and C21 demonstrated noteworthy affinity towards the receptor with scores of −7.1 kcal/mol and −7 kcal/mol, respectively, compared to the Lapatinib binding affinity of −10.6 kcal/mol. Also, C19 demonstrated the highest activity against BCL-2, showing an affinity of −6.7 kcal/mol compared to the standard Lapatinib affinity of −8.4 kcal/mol. Meanwhile, C2, C4, C21, and C23 exhibited promising affinities of −6.3, −6.4, −6.3, and −6.6 kcal/mol towards the receptor. The C19 suppressed the binding affinity of standard Diclofenac against COX-2 with a binding score of −7.9 kcal/mol, whereas the Diclofenac showed −7.8 kcal/mol. Moreover, C23 manifested equal binding affinity as standard. Promising binding affinities were also observed for C9, C14, C18, and C21 with affinities of −7.3, −7.2, −7.4, and −7.2 kcal/mol, respectively. Finally, in the case of TNF-α, C19 exhibited a very potential binding effect with an affinity of −7.4 kcal/mol, which easily suppressed the affinity of Diclofenac (−7.1 kcal/mol). Also, C8, C9, C18, and C23 registered affinities of −6.5, −6.7, −6.3, and −6.5 kcal/mol, respectively. The ADME/T analysis of selected compounds with prospective value in molecular docking simulation studies has been subjected to ADME/T analysis to have a prior safety and efficacy profile. The compounds also show promising safety and efficacy backgrounds in this computer-based analysis.

**TABLE 6:** Binding affinities of the identified compounds and standards against ten receptors representing antidiarrheal, antimicrobial, antioxidant, cytotoxic, and analgesic activities.

Fractions	Compound serial	Compound name	PubChem CID	MW (g/mol)	Targets									
					Antidiarrheal		Antimicrobial		Antioxidant		Cytotoxic		Analgesic	
					Kappa opiod receptor (KOR)	Human delta opiod receptor (DOR)	Beta-ketoaryl-ACP syntase 3 (KAS)	Dihydrofolate reductase (DHFR)	Glutathion reductase (GLR)	Urate oixidase (URO)	Epidermal growth factor receptor (EGFR]	B-cell lymphoma 2 (BCL-2)	Cyclooxygenase- 2 (COX-2)	Tumor necrosis factor alpha (TNF-α)
DCMSF	1	Pentanoic acid, 4-nitro-, methyl ester	229,650	161.16	−3.6	−5.3	−5	−4.6	−5.7	−4.3	−5.1	−4.8	−5.2	−4.8
DCMSF	2	Azulene	9231	128.169	−5.3	−7	−5.9	−6	−5.8	−4.9	−6.5	−6.3	−7.1	−5.9
DCMSF, HSF, AQSF	3	9-Methoxybicyclo[6.1.0]nona-2,4,6-triene	5,370,526	148.2	−4.9	−6.6	−5.5	−5.4	−5.5	−4.7	−5.6	−6.2	−6.6	−5.6
DCMSF	4	Benzenemethanol, .alpha.-methyl-.alpha.-propyl-	138,214	164.24	−5	−7	−5.6	−5.4	−5.8	−4.9	−6.6	−6.1	−6.3	−5.9
DCMSF	5	3-Tetradecene, (Z)-	5,362,709	196.37	−4.9	−6	−5.4	−4.6	−4.3	−3.9	−5.9	−5	−6.1	−5
DCMSF, EASF, AQSF	6	Estragole	8815	148.2	−5.1	−6.4	−5.6	−5.3	−5.5	−4.5	−5.7	−5.4	−5.7	−5.1
DCMSF	7	Dodecane, 2,6,11-trimethyl-	35,768	212.41	−5.4	−6.7	−5.2	−4.9	−5	−4.4	−6.2	−5.8	−6.8	−5.6
DCMSF, EASF	8	Phenol, 2,6-bis(1,1-dimethylethyl)-	31,405	206.32	−5.7	−7	−5.8	−6.1	−5.6	−5.7	−6.7	−6.2	−6.8	−6.5
DCMSF, EASF, HSF, AQSF	9	Phenol, 3,5-bis(1,1-dimethylethyl)-	70,825	206.32	−5.8	−6.5	−6	−6.4	−5.8	−5.7	−6.3	−6.4	−7.3	−6.7
DCMSF	10	1-Undecene	13,190	154.29	−4.7	−5.4	−5	−4	−4.4	−3.5	−5	−5	−5.4	−4.6
DCMSF	11	3-Hexadecene, (Z)-	5,364,494	224.42	−5.2	−5.8	−5.8	−4.8	−4.7	−4.3	−6.1	−5.4	−6.5	−5
DCMSF	12	Pentafluoropropionic acid, decyl ester	536,957	304.3	−5.2	−6.7	6.3	−5.7	−6.2	−5.2	−7.1	−6.1	−6.9	−5.8
DCMSF	13	Undecanoic acid, 10-methyl-, methyl ester	554,144	214.34	−5	−6.3	−5.4	−4.8	−5.6	−4.4	−5.9	−5.1	−6.1	−5
DCMSF	14	9,12-Octadecadienoic acid, methyl ester, (E,E)-	5,362,793	294.5	−5.7	−6.4	−6	−5.5	−5.9	−4.8	−6.5	−5.6	−7.2	−5.5
DCMSF	15	9-Octadecenoic acid (Z)-, methyl ester	5,364,509	296.5	−5.6	−6.5	−5.2	−5.2	−5.2	−4.7	−6.5	−5.6	−7	−5.2
DCMSF	16	13-Docosenoic acid, methyl ester, (Z)-	5,364,423	352.6	−4.8	−6.1	−5.7	−5.5	−5.4	−4.9	−6.4	−5.5	−6.7	−5.8
DCMSF	17	15-Tetracosenoic acid, methyl ester, (Z)-	5,364,841	380.6	−4.7	−6.9	−6	−5.4	−5.3	−4.9	−6.3	−5.8	−6.9	−5.8
DCMSF	18	Di-n-octyl phthalate	8346	390.6	−4.9	−6.4	−6.1	−6.1	−6.9	−5.4	−5.8	−6	−7.4	−6.3
DCMSF	19	4,6-Bis(1,1-dimethylethyl)-1,1′-biphenyl-2-ol	13,543,504	282.4	−6.9	−6.8	−6.8	−7.4	−7	−6.8	−7.8	−6.7	−7.9	−7.4
DCMSF	20	Cyclotetrasiloxane, octamethyl-	11,169	296.61	−1.2	−1.4	−1.6	−1.4	−1.7	−1.8	−1.7	−1.8	−1.5	−1.1
EASF, AQSF	21	Heptadecane, 2,6,10,15-tetramethyl-	41,209	296.6	−5.3	−6.4	−6.3	−5.3	−6.1	−4.9	−7	−6.3	−7.2	−5.8
EASF, AQSF	22	Tetracosane	12,592	338.7	−5.1	−6.4	−4.9	−4.8	−4.7	−4.3	−6.2	−5.7	−7	−5.1
EASF	23	Diisooctyl phthalate	33,934	390.6	−4.7	−6.7	−6.4	−6.3	−7.3	−5.3	−7.4	−6.6	−7.8	−6.5
HSF	24	9-Oxabicyclo[6.1.0]nonan-4-ol	273,831	142.2	−3.9	−5.8	−5.1	−5.1	−5.6	−4.8	−5.2	−4.9	−5.7	−5.3
HSF	25	Cyclohexane, 1,1-dimethoxy-	13,616	144.21	−3.9	−5.7	−4.5	−4.6	−5.1	−4.3	−5.1	−5	−5	−4.8
HSF	26	Epoxy-linalooloxide	537,453	186.25	−4.1	−6	−4.9	−5.3	−6	−4.8	−5.6	−5.4	−5.7	−5.1
HSF	27	n-Tridecan-1-ol	8207	200.36	−4.9	−5.7	−5.2	−4.5	−4.9	−4.2	−5.6	−4.9	−5.8	−4.6
HSF	28	1-Tetradecene	14,260	196.37	−4.9	−5.6	−5	−4.3	−4.5	−3.9	−5.5	−4.9	−6	−4.9
AQSF	29	Undecane, 5,7-dimethyl-	519,405	184.36	−4.8	−6.4	−5.5	−4.9	−5.1	−4.4	−5.8	−5.5	−6.1	−5
AQSF	30	Dodecane	8182	170.33	−4.8	−5.5	−4.7	−4.2	−4	−3.8	−5	−4.8	−5.4	−4.4
AQSF	31	Octane, 2,3,7-trimethyl-	43,867	156.31	−4.8	−6.3	−5.1	−5	−4.8	−4.5	−5.5	−5.4	−5.9	−5.2
AQSF	32	Hexane, 3,3-dimethyl-	11,233	114.23	−4.1	−5.1	−4.5	−4.1	−4	−3.6	−4.5	−4.6	−4.7	−4.3
AQSF	33	Dodecane, 2,6,10-trimethyl-	19,773	212.41	−5.4	−6.8	−5.9	−4.8	−5.3	−4.8	−6	−5.8	−6.9	−5.8
AQSF	34	Dodecane, 4-methyl-	521,958	184.36	−4.8	−6.1	−5.3	−4.7	−4.9	−4	−5.4	−5	−5.7	−4.7
AQSF	35	1-Octanol, 2-butyl-	19,800	186.33	−4.5	−5.9	−5.3	−4.6	−4.5	−4.1	−5.5	−5.1	−5.7	−5.3
AQSF	36	Decane, 4-ethyl-	519,256	170.33	−4.5	−5.9	−5.3	−4.4	−4.3	−3.9	−5.1	−5	−5.5	−4.8
AQSF	37	Decane, 2,3,5,8-tetramethyl-	545,611	198.39	−5.4	−7	−5.7	−5	−5.4	−4.5	−6.3	−6.1	−6.4	−5.5
AQSF	38	Tridecane	12,388	184.36	−4.7	−5.7	−5	−4.3	−4.6	−4	−5.4	−4.9	−5.6	−4.5
AQSF	39	Pentadecane	12,391	212.41	−4.9	−6	−5.4	−4.4	−4.3	−3.9	−5.6	−5.2	−6.1	−4.7
AQSF	40	Octadecane	11,635	254.5	−5.2	−5.6	−5.6	−4.8	−4.7	−4	−5.9	−5.1	−6.5	−5
AQSF	41	Tetradecane, 4-methyl-	520,179	212.41	−4.9	−6.2	−5.7	−4.8	−4.5	−4.1	−5.6	−5	−6.3	−4.8
AQSF	42	Heneicosane	12,403	296.6	−5	−5.9	−5.4	−4.9	−4.8	−4.2	−6.1	−5.5	−6.5	−5.4
AQSF	43	Tetradecane, 4,11-dimethyl-	108,309	226.44	−5.4	−6.5	−5.6	−5	−4.7	−4.3	−5.9	−5.1	−6.6	−5
AQSF	44	2,3-Dimethyldodecane	521,959	198.39	−5.1	−6.4	−5.8	−5.2	−4.3	−4.2	−5.7	−5.2	−6	−5
AQSF	45	Pentadecane, 2,6,10-trimethyl-	19,775	254.5	−5.4	−6.9	−6.1	−5.3	−4.9	−4.6	−6.7	−5.5	−6.8	−5.6
AQSF	46	Hexadecane	11,006	226.44	−4.9	−5.8	−5.4	−4.5	−4.3	−4	−5.8	−5.1	−6.4	−4.8
AQSF	47	Nonadecane	12,401	268.5	−5.1	−5.9	−5.4	−4.9	−4.3	−4.6	−5.8	−5.4	−6.5	−5
AQSF	48	Eicosane	8222	282.5	−4.6	−6.3	−5.5	−4.9	−4.7	−4.3	−5.8	−5.3	−6.6	−5.2
Standards		Loperamide	3955	477	−7.7	−8.9	-	-	-	-	-	-	-	-
		Amoxicillin	33,613	365.4	-	-	−7.1	−7.6	-	-	-	-	-	-
		Ascorbic acid	54,670,067	176.12	-	-	-	-	−6.4	−5.3	-	-	-	-
		Lapatinib	208,908	581.1	-	-	-	-	-	-	−10.6	−8.4	-	-
		Diclofenac	3033	296.1	-	-	-	-	-	-	-	-	−7.8	−7.1

## Discussion

### GC-MS and phytochemical analysis

Vegetables abound in a multitude of vitamins, minerals, nutritive phytochemicals or secondary metabolites that carry diverse phototherapeutic properties (Wolfender et al., 2011). In both modern and folk medicine systems, the secondary metabolites of vegetables, namely, alkaloids, fatty acids, flavonoids, phenolic compounds, tannins and so on, have provided ethnopharmacological actions such as, analgesic, antidiarrheal, antimicrobial, and antioxidant activities (Alam et al., 2021b; Nakaziba et al., 2021). Based on previous research, 80% of its 122 drug moieties derived from phyto sources closely aligned with their original ethnopharmacological purposes (Nakaziba et al., 2021). Consequently, vegetables deciphering therapeutic potentials can open a new well-spring for novel drug discovery or enhance existing therapeutics.

Plant extracts often consist of complex mixtures of secondary metabolites obtained from plants, animals, and microbes. These extracts usually comprised 10 to 60 metabolites with different quantities, but their biological properties are mainly attributed to two to four significant molecules (Rahman et al., 2013). Through the analysis of the chemical composition and arrangement of samples, numerous biological capabilities can be revealed inside extracts from medicinal plants. Remarkably, there is a conspicuous lack of published studies employing GC-MS/MS to characterize bioactive compounds in the *C. affinis* plant. To fill this gap, a well-organized inquiry was conducted, which involved the use of GC–MS/MS analysis. Phenolic compound 3,5-bis(1,1-dimethylethyl), was notably abundant in all extract fractions except for EA. Conversely, its analogous phenolic compound, 2,6-bis(1,1-dimethylethyl), demonstrated a substantial presence, accounting for 63.907%, specifically within the EA fraction (Table 1). Research showed that phenol, 3,5-bis(1,1-dimethylethyl)- also known as 3,5-di-tert-butylphenol demonstrated antifungal effects on *Candida* strains by inhibiting biofilm formation and affecting planktonic cell viability. It caused structural alterations in free-floating and surface-attached cells, notably impacting cell membrane integrity. Additionally, 3,5-DTB exhibited synergism with sodium dodecyl sulfate (SDS), disrupting membrane integrity further. The chemical also generated reactive oxygen species (ROS) in *Candida*, adding to its anti-biofilm function (Vijayakumar and MuhilVannan, 2021). However, this compound and its equivalent 2,6-ditert-butylphenol or Phenol, 2,6-bis (1,1-dimethyl ethyl)- has been documented to have several activities, including antioxidant, cytotoxic, insecticidal, and nematicidal. antibacterial, and antiviral (Zhao et al., 2020). A considerable amount of fatty acid, especially methyl esters, was observed from different plant fractions (Table-). 9-Octadecenoic acid (Z)- exhibits notable efficacy as a potent antibacterial and antiviral compound, showcasing promising capabilities in combating microbial infections (Hadi and Hussein, 2016). Estragole is commonly used in food flavoring, and while studies indicate its potential carcinogenicity, controversial mutagenicity results exist, with its key biological activity linked to the formation of hepatic DNA adducts by metabolites (De Vincenzi et al., 2000). Diisooctyl phthalate, a derivative of phthalic acid esters, belongs to a group of lipophilic chemicals known as plasticizers. These metabolites are extensively utilized to enhance mechanical extensibility and flexibility in various products. Additionally, they are recognized for exhibiting diverse biological activities, including allelopathic, antimicrobial, and insecticidal properties (Huang et al., 2021).

Flavonoids are a class of organic chemicals with a wide range of phenolic structures. They are commonly found in many natural sources, such as fruits, vegetables, grains, bark, roots, stems, flowers, tea, and wine. These appeared to have several bioactivities, including anti-diabetic, anti-inflammatory, antibacterial, antioxidant, antiviral, cytotoxic, and lipid-lowering activities (Hasnat et al., 2024). The apparent reason for the antioxidant, anti-inflammatory, antibacterial, and cytotoxic activities in leaf extract of *C. affinis* is accountable owing to the presence of flavonoids found throughout the botanical herb (Table 2).

Antioxidants play a vital role in neutralizing reactive oxygen and nitrogen species generated by the human immune system, thereby mitigating oxidative stress. This stress, if left unaddressed, has the potential to harm cells and tissues by causing damage to DNA, lipids, and proteins (Rajlic et al., 2023). Several studies reported eicosane, 15-tetracosenoic acid derivatives, and phenol derivatives metabolites to exhibit antioxidant activity (Wojdyło et al., 2007; Medaowe et al., 2020; Balachandran et al., 2023). Interestingly, GC-MS data from *C. affinis* fractions also demonstrated the presence of these metabolites. Quantitative analysis of *C. affinis* AQSF showed a maximum total phenolic content of 57.23 mg GAE/g, among other things. Furthermore, AQSF demonstrated remarkable antioxidant activity in the DPPH scavenging assessment, with an IC_50_ value of 29.40 μg/mL. The different fractions also deciphered promising antioxidant attributes, which may validate the presence of antioxidant metabolites in GC-MS data.

### Biological activities

The analgesics alleviate pain sensation via several mechanisms, including inhibition of cyclooxygenase enzymes (COX-1 and COX-2), inhibition of activated transcription factors, and binding with receptors which increase transcription of anti-inflammatory proteins. Anti-inflammatory agents also relieve pain associated with inflammatory conditions by interfering with the biosynthesis of inflammatory mediators (Alam et al., 2020; Phull et al., 2022). Azulene, eicosane, heneicosane, and some phenolic compounds found in *C. affinis* extractives have been reported to exhibit substantial analgesic and anti-inflammatory activities (Ambriz-Pérez et al., 2016; Okechukwu, 2020; Mousa et al., 2022). Probably for this reason, during the analgesic assay of *C. affinis* fractionated extracts, notable analgesic actions were observed ranging from 16.28% to 52.33% compared to standard diclofenac sodium with 77.91% inhibition of writhing. Furthermore, cancer is a widespread health condition characterized by unregulated cell proliferation and the capacity to infiltrate or inflame surrounding tissues. Cytotoxic compounds can halt the expansion of cancer cells (Guzow-Krzemińska et al., 2019). Estragole and Tetracosane are the metabolites found in *C. affinis* as per GC-MS data and possess cytotoxic activities (Uddin et al., 2012; Andrade et al., 2015). In quantitative cytotoxicity analysis of different *C. affinis* fractions, DCMSF (LC_50_ 3.21) and AQSF (LC_50_ 1.36) exerted promising cytotoxic properties, among others, followed by EASF and HSF when compared to vincristine sulfate (LC_50_ 0.451). The cytotoxic activities of these fractions may occur due to the presence of above-mentioned metabolites. An antidiarrheal compound is a substance or medication that exerts its effects through mechanisms to mitigate or prevent diarrhea. These metabolites typically target various mechanisms, such as slowing down bowel movements, reducing intestinal inflammation, augmenting water absorption or addressing the underlying cause of diarrhea to restore normal bowel function (Beserra et al., 2016). The DCMSF of *C. affinis* exerted significant antidiarrheal activity with 51.16% diarrheal inhibition followed by AQSF, HSF, and EASF, compared to 67.44% diarrheal inhibition by standard loperamide. The presence of azulene in DCM soluble fraction can be the underlying cause of such activity, as azulene has been reported to have a promising antidiarrheal and anti-inflammatory potential in previous (Aziz et al., 2011; Bakun et al., 2021) Bacteria inherently possess a genetic inclination to evolve resistance, underscoring the need for the continual development of novel antimicrobial drugs to combat a wide range of microbes (Nascimento et al., 2000). The antimicrobials target diverse mechanisms of action such as disruption of microbial cell wall synthesis, the intervention of key enzymatic pathways, and interference with the production of genetic materials and proteins (Epand and Vogel, 1999; Ghannoum and Rice, 1999). The GC-MS analysis of *C. affinis* fractionated extracts has exerted the presence of eicosane, heneicosane, estragole, and some other phenolic compounds, which show significant antifungal and antimicrobial activities according to previous studies (Park et al., 2001; Donati et al., 2015; Vanitha et al., 2020; Octarya et al., 2021)AQSF and DCMSF showed relatively higher ZOI, followed by DCMSF against both gram-positive and gram-negative bacteria. The presence of the antimicrobial estragole and phenolic compounds in these fractions is the reason for such attributes. Besides, the antifungal compounds eicosane and heneicosane in AQSF and EASF made them exhibit prominent antifungal properties.

### Molecular docking and ADME/T study

**FIGURE 4:**
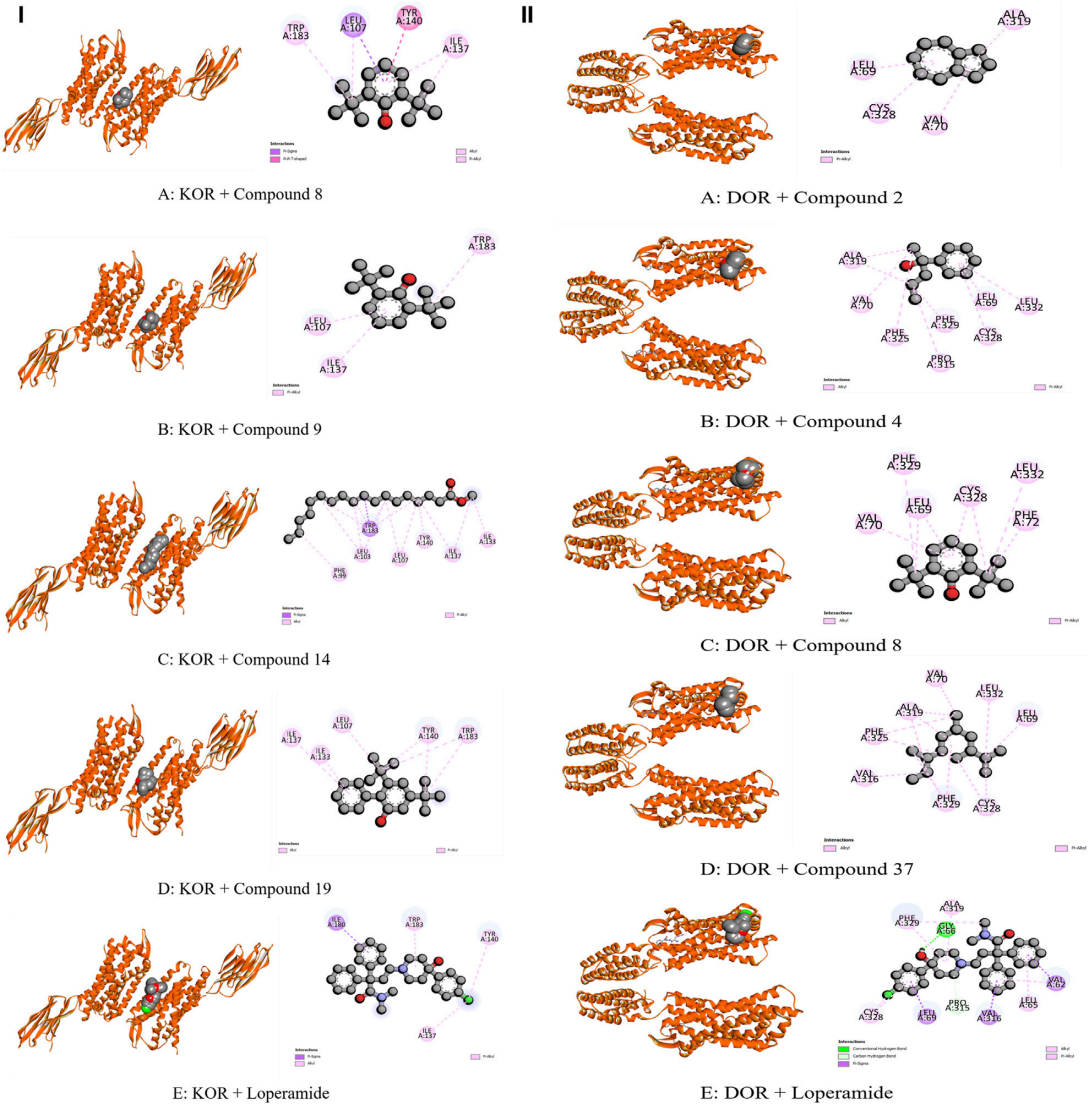
Molecular Interactions of Phytochemicals with KOR and DOR Enzymes: **(I)** Graphical representation of the molecular interactions of the most prominent phytocompounds with the KOR enzyme in 3D visualization (Compound 8 = A, Compound 9 = B, Compound 14 = C, Compound 19 = D, and Standard Loperamide = E). **(II)** Graphical representation of the molecular interactions of the most prominent phytocompounds with the DOR enzyme in 3D visualization (Compound 2 = A, Compound 4 = B, Compound 8 = C, Compound 37 = D, and Standard Loperamide = E).

**FIGURE 5:**
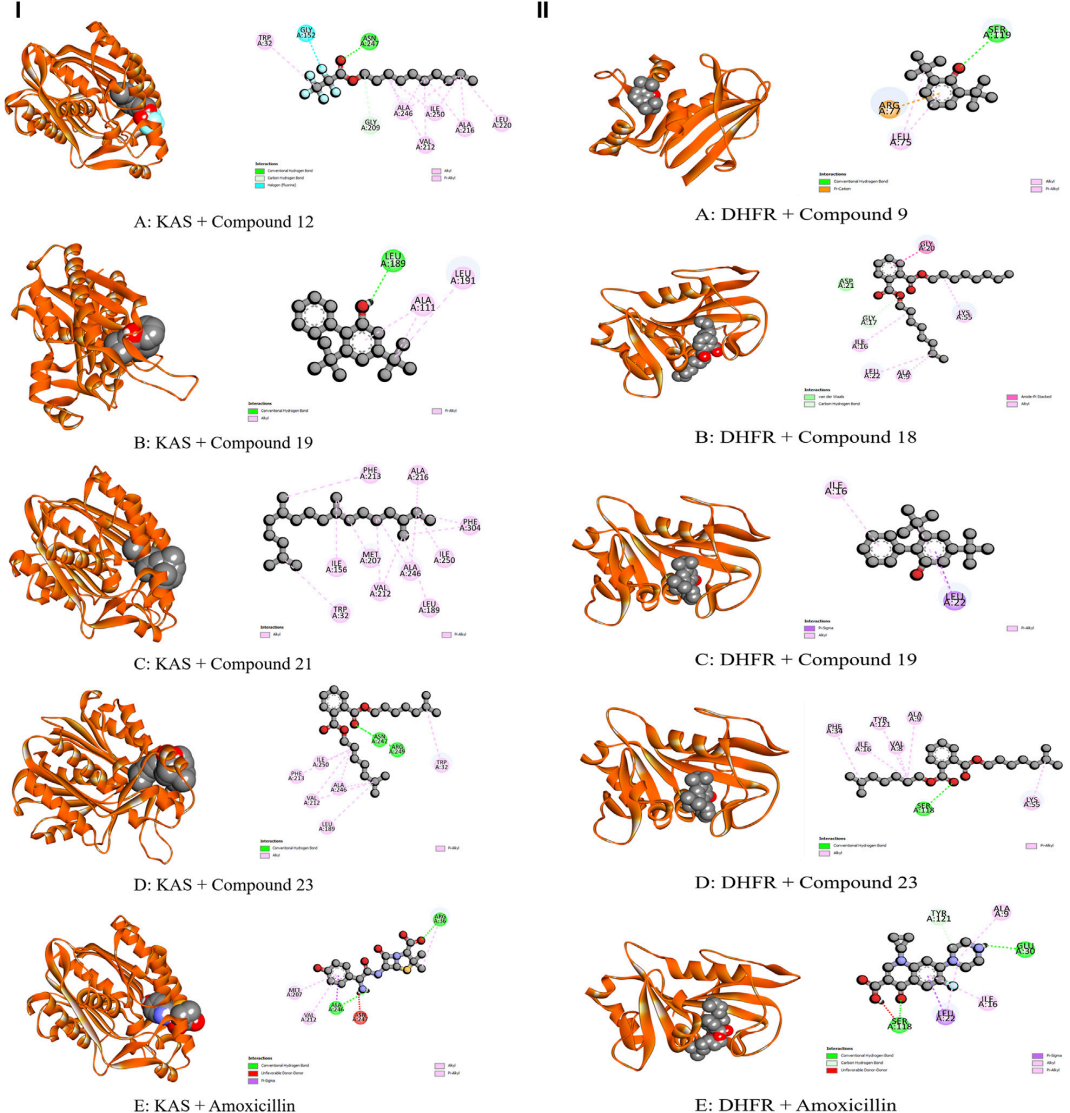
Molecular Interactions of Phytochemicals with KAS and DHFR Enzymes: **(I)** Graphical representation of the molecular interactions of the most prominent phytocompounds with the KAS enzyme with 3D visualization (Compound 12 = A, Compound 19 = B, Compound 21 = C, Compound 23 = D, and Standard Amoxicillin = E). **(II)** Graphical representation of the molecular interactions of the most prominent phytocompounds with the DHFR enzyme with 3D visualization (Compound 9 = A, Compound 18 = B, Compound 19 = C, Compound 23 = D, and Standard Amoxicillin = E).

**FIGURE 6:**
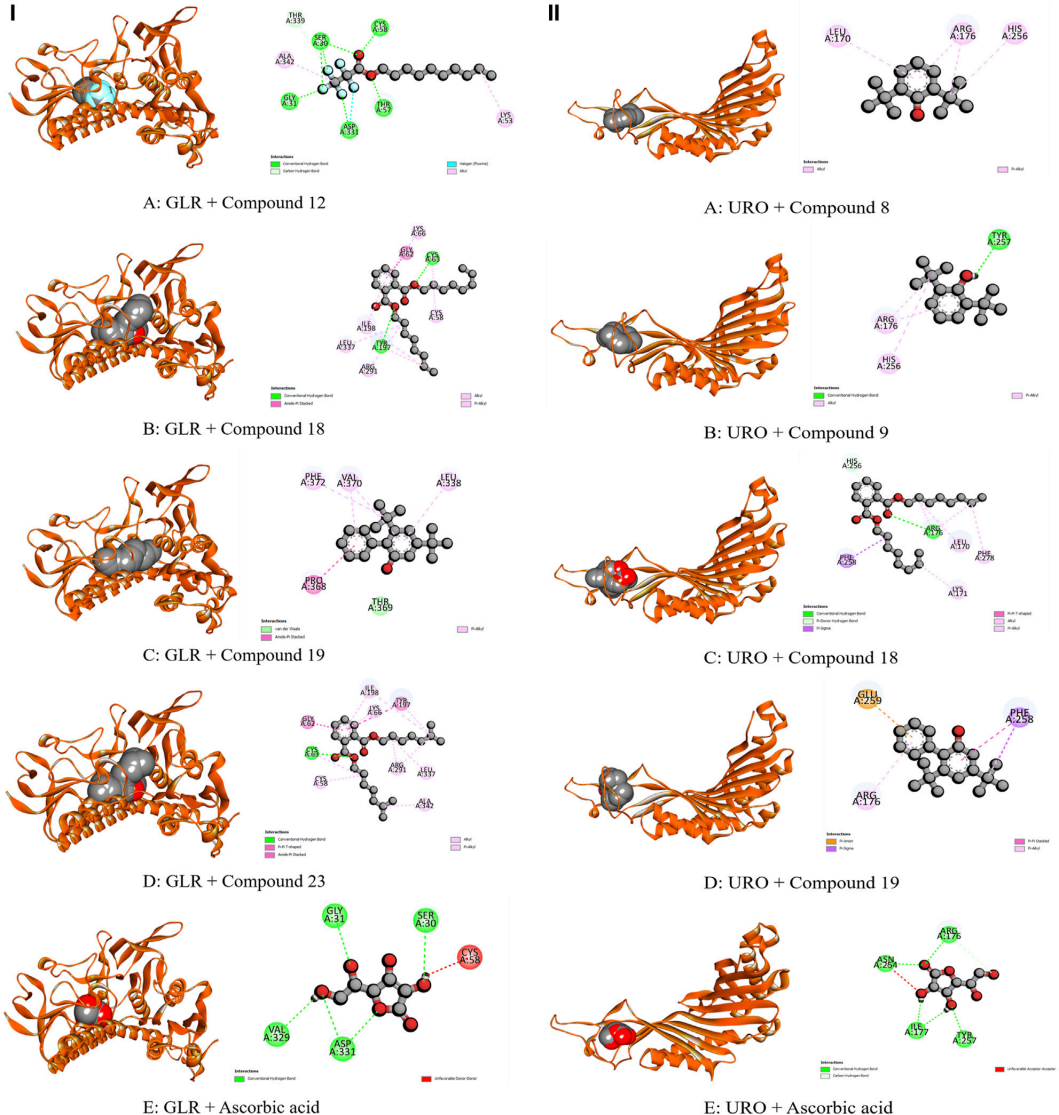
Molecular Interactions of Phytochemicals with GLR and URO Enzymes: **(I)** Graphical representation of the molecular interactions of the most prominent phytocompounds with the GLR enzyme with 3D visualization (Compound 12 = A, Compound 18 = B, Compound 19 = C, Compound 23 = D, and Standard Ascorbic acid = E). **(II)** Graphical representation of the molecular interactions of the most prominent phytocompounds with the URO enzyme with 3D visualization (Compound 8 = A, Compound 9 = B, Compound 18 = C, Compound 19 = D, and Standard Ascorbic acid = E).

The modulation of gastrointestinal (GI) signaling in the human body involves opioid receptors, namely, µ, ƙ, and δ receptors. These receptors exercise their impact by limiting the activity of enteric nerves, reducing the discharge of neurotransmitters, and reducing the strength of excitatory and inhibitory motor pathways. Consequently, this cascade of effects leads to delayed colonic transit, diminished excitability of enteric nerves, and modifications in secretion and fluid transport. Ultimately, these alterations culminate in changes to motility and stool consistency (Pannemans and Corsetti, 2018). Sevaral compounds from *C. affinis* have displayed noteworthy potential against KOR, with C19 exhibiting the highest affinity (−6.9 kcal/mol). It forms bonds with five amino acids through alkyl interactions. In contrast, C9 establishes pi-alkyl bonds, resulting in a binding score of −5.8 kcal/mol. This is compared to the standard Loperamide, which forms a single pi-alkyl bond and two alkyl bonds, achieving a binding score of −7.7 kcal/mol (Table 7; Figure 4). Meanwhile, C2, C4, C8, and C37 demonstrated significant activity against DOR, showing binding affinities of −7 kcal/mol. They connect to the receptor through four pi-alkyl, eight alkyl, six alkyl, and eight alkyl bonds, respectively. The standard Loperamide binds to this receptor through four alkyls, three pi-sigma, and a single carbon-hydrogen bond, forming a more favorable binding score of −8.9 kcal/mol (Table 7; Figure 4). Beta-ketoacyl-ACP synthase 3 is an essential enzyme that plays a key part in antimicrobial activities in microbial species. It contributes actively to mycolic acid synthesis, an essential component of the microbial cell wall, by participating in the last steps of FAS-II condensation and extension. Mycolic acid biosynthesis may be hampered by blocking the activity of this enzyme, making it a viable target for antimicrobial therapies against such pathogenic diseases (Singh et al., 2011). According to the computational docking analysis, C19 demonstrated a binding affinity of −6.8 kcal/mol against KAS, nearly matching the −7.1 kcal/mol binding score of the standard Amoxicillin. C19 established connections through two alkyl bonds and a conventional hydrogen bond. Conversely, the standard Amoxicillin interacted with KAS through two conventional hydrogen bonds, a single alkyl bond, pi-sigma interaction, and unfavorable donor-donor interactions. In addition, C12, C21, and C23 also exhibited potential affinity towards the receptor (Table 7; Figure 5). The bacterial dihydrofolate reductase (DHFR) is essential in producing thymidylate, making it a highly potential candidate for the treatment of infections. Inhibitors of DHFR can lead to bacterial death, providing a potential avenue for addressing microbial infections (He et al., 2020). Furthermore, disturbances in the folate pathway, facilitated by DHFR, might lead to unregulated cell proliferation, affecting cellular proliferation and development in different cancers (Kodidela et al., 2016). Ciprofloxacin formed two conventional hydrogen bonds, two alkyl bonds, and a single carbon-hydrogen and pi-sigma bond when interacting with DHFR. This interaction resulted in a binding score of −7.6 kcal/mol. In contrast, C19 demonstrated a highly promising affinity with a score of −7.4 kcal/mol, forming a single pi-sigma and alkyl bonds. Conversely, C8, C9, C18, and C23 displayed affinities lower than −6 kcal/mol, establishing 2, 3, 4, and 2 different types of interactions, respectively (Table 7; Figure 5). Glutathione reductase plays a crucial role in maintaining the antioxidant activity of the tripeptide glutathione. Catalyzing the regeneration of its reduced form from the oxidized state ensures the balance of the cellular redox state. This enzyme, featuring flavin adenine dinucleotide as a redox-active group, utilizes nicotinamide adenine dinucleotide phosphate (NADPH) as a specific reductant to convert glutathione disulfide (GSSG) back to its reduced state. The specificity of glutathione reductase for NADPH and its selective activity with certain substrates highlights its significance in preserving the cellular antioxidant defense system (Mannervik, 1999). Regarding GLR, C23, C19, and C18 exhibited inhibitory effects on the standard Ascorbic acid binding affinity, which was measured at −6.4 kcal/mol. Specifically, C23 formed a binding score of −7.3 kcal/mol by engaging a conventional hydrogen bond, two pi-pi bonds, and six alkyl bonds. In contrast, C19 demonstrated a binding score of −7 kcal/mol by establishing three pi-alkyl bonds and single van der Waals and aide pi-stacked interactions. Additionally, C18 bound with a score of −6.9 kcal/mol, utilizing five alkyl bonds, two conventional hydrogen bonds, and an amide pi-stacked bond. In comparison, Ascorbic acid displayed binding through four conventional hydrogen bonds and an unfavorable donor-donor interaction (Table 7; Figure 6). The uric acid degrading URO, also known as uricase, converts uric acid into 5-hydroxy isourate and H_2_O_2_, amplifying oxidant stress and its associated disorders (Cleveland et al., 2009). By inhibiting URO, certain identified metabolites demonstrate potential antioxidant activity. Notably, C19, C8, C9, and C18 exhibited a surprising enhancement in receptor affinity, with binding scores of −6.8, −5.7, −6.7, and −5.4 kcal/mol, respectively, surpassing the standard affinity of −5.3 kcal/mol. These improved affinities resulted from specific interactions between the ligands and receptors. For instance, C19 engaged in single Pi-alkyl, Pi-sigma, and Pi-Donor Hydrogen interactions, while C8 formed three alkyl bonds. C9 demonstrated two Alkyl bonds and a conventional hydrogen bond, and C18 interacted through three alkyl bonds, along with single pi-sigma, pi-donor hydrogen, and conventional hydrogen bonds. Notably, C18’s binding pattern surpassed that of Ascorbic acid, which formed four conventional hydrogen bonds (Table 7; Figure 6).

**TABLE 7:** Bond and binding site of highly active compounds against different targets including KOR, DOR, KAS, DHFR, GLR, URO, BCL-2, EGFR, COX-2, and TNF-α.

Receptor	Compounds	Biniding affinites (kcal/mol)	Bond type	Amino acids
KOR	C8	−5.7	Pi- sigma	Leu 107
			Pi-Pi	Tyr 140
			Alkyl	Ile 137, Trp 183
	C9	−5.8	Pi-Alkyl	Leu 107, Ile 137, TRP 183
	C14	−5.7	Pi- sigma	Trp 183
			Alkyl	Phe 99, Leu 103, Leu 107, Ile 133, Ile 137, Tyr 140
	C19	−6.9	Alkyl	Leu 107, Ile 133, Ile 137, Tyr 140, TRP 183
	Loperamide	−7.7	Pi- Sigma	Ile 180
			Alkyl	Tyr 140, Trp 183
DOR	C2	−7	Pi- Alkyl	Leu 69, Val 70, Ala 319, Cys 328
	C4	−7	Alkyl	Leu 69, Val 70, Pro 315, Ala 319, Phe 325, Cys 328, Phe 329, Leu 332
	C8	−7	Alkyl	Leu 69, Val 70, Phe 72, Cys 328, Phe 329, Leu 332
	C37	−7	Alkyl	Leu 69, Val 70, Val 316, Ala 319, Phe 325, Cys 328, Phe 329, Leu 332
	Loperamide	−8.9	Alkyl	Leu 69, Ala 319, Cys 328, Phe 329
			Pi- Sigma	Val 62, Leu 69, Val 316
			Carbon-hydrogen bond	Pro 315
			Conventional Hydrogen Bond	Gly 66
KAS	C12	−6.3	Alkyl	Trp 32, Val 212, Ala 216, Leu 220, Ala 246, Ile 250
			Carbon-hydrogen bond	Gly 209
			Conventional Hydrogen Bond	Asn 247
			Halogen(Fluorine)	Gly 152
	C19	−6.8	Conventional Hydrogen Bond	Leu 189
			Alkyl	Ala 111, Leu 191
	C21	−6.3	Alkyl	Trp 32, Ile: 156, Leu 189, Met 207, Val 212, Phe 213, Ala 216, Ala 246, Ile 250, Phe 304
	C23	−6.4	Conventional Hydrogen Bond	Asn 247, Arg 249
			Alkyl	Trp 32, Leu 189, Val 212, Phe 213, Ala 246, Ile 250
	Amoxicillin	−7.1	Conventional Hydrogen Bond	Arg 36, Ala 246
			Alkyl	Val 212
			Pi- Sigma	Met 207
			Unfavorable Donor- Donor	Asn A:247
DHFR	C8	−6.1	Pi-Pi T-shaped	Tyr 121
			Pi-Alkyl	Ile 16, Leu 22
	C9	−6.4	Conventional Hydrogen Bond	Ser 119
			Alkyl	Leu 75
			Pi- Carbon	Arg 77
	C18	−6.1	Van der waals	Asp 21
			Carbon-hydrogen bond	Gly 17
			Amide-Pi-Stacked	Gly 20
			Alkyl	Ala 9, Ile 16, Lys 55
	C19	−7.4	Pi- Sigma	Leu 22
			Alkyl	Ile 16
	C23	−6.3	Conventional Hydrogen Bond	Ser 118
			Alkyl	Val 8, Ala 9, Ile 16, Phe 34, Lys 55, Tyr 121
	Ciprofloxacin	−7.6	Conventional Hydrogen Bond	Glu 30, Ser 118
			Carbon-hydrogen bond	Tyr 121
			Pi- Sigma	Leu 22
			Alkyl	Ala 9, Ile 16
GLR	C12	−6.2	Alkyl	Lys 53, Ala 342
			Carbon-hydrogen bond	Thr 339
			Conventional Hydrogen Bond	Ser 30, Gly 31, Thr 57, Cys 58, Asp 331
	C18	−6.9	Alkyl	Cys 58, Lys 66, Ile 198, Arg 291, Leu 337
			Conventional Hydrogen Bond	Cys 63, Tyr 197
			Amide-Pi-Stacked	Gly 62
	C19	−7	Van der waals	Thr 369
			Amide-Pi-Stacked	Pro 368
			Pi- Alkyl	Leu 338, Val 370, Phe 372
	C23	−7.3	Conventional Hydrogen Bond	Cys 63
			Pi-Pi	Gly 62, Tyr 197
			Alkyl	Cys 58, Lys 66, Ile 198, Arg 291, Leu 337, Ala 291
	Ascorbic acid	−6.4	Unfavorable Donor- Donor	Cys 58
			Conventional Hydrogen Bond	Ser 30, Gly 31, Val 329, Asp 331
URO	C8	−5.7	Alkyl	Leu 170, Arg 176, His 256
	C9	−5.7	Conventional Hydrogen Bond	Tyr 257
			Alkyl	Arg 176, His 256
	C18	−5.4	Conventional Hydrogen Bond	Arg 176
			Alkyl	Leu 170, Lys 171, Phe 278
			Pi- Sigma	Phe 258
			Pi- Donor Hydrogen Bond	His 256
	C19	−6.8	Pi-Alkyl	Arg 176
			Pi- Sigma	phe 258
			Pi- Anion	Glu 259
	Ascorbic acid	−5.3	Conventional Hydrogen Bond	Arg 176, Ile 177, Asn 254, Tyr 257
BCL-2	C2	−6.3	Pi-Pi T-shaped	Phe 101
			Pi-Alkyl	Met 112, Ala 146
	C9	−6.4	Conventional Hydrogen Bond	Asp 108
			Pi- Sigma	Met 112
			Alkyl	Phe 101
	C19	−6.7	Pi-Alkyl	Phe 101, Leu 134
	C21	−6.3	Alkyl	Phe 101, Tyr 105, Phe 109, Met 112, Val 130, Leu 134, Ala 146
	C23	−6.6	Pi- Sigma	Tyr 105, Leu 134
			Alkyl	Phe 101, Met 112, Ala 146
	Lapatinib	−8.4	Pi- Action	Asp 108
			Pi-Pi T-shaped	Tyr 105
			Alkyl	Met 112, Leu 134
EGFR	C12	−7.1	Conventional Hydrogen Bond	Thr 790, Asp 855
			Halogen(Fluorine)	Met 766, Cys 775, Arg 776, Thr 854, Phe 856
			Alkyl	Leu 718, Val 726, Ala 743, Lys 745, Leu 844
	C19	−7.8	Unfavorable Aceptor- Aceptor	Asp 855
			Pi- Sigma	Val 726
			Alkyl	Leu718, Cys 797, Leu 844
	C21	−7	Pi- Sigma	Phe 856
			Alkyl	Leu 718, Val 726, Ala 743, Lys 745, Met 766, Cys 775, Leu 777, Leu 788, Leu 844
	C23	−7.4	Conventional Hydrogen Bond	Cys 797
			Pi- Sigma	Leu 844
			Alkyl	Leu 718, VAl 726, Ala 743, Lys 745, Tyr 998, Leu 1001, Met 1002
	Lapatinib	−10.6	Conventional Hydrogen Bond	Thr 790
			Pi- Carbon	Lys 745
			Halogen(Fluorine)	Cys 775, Arg 776
			Carbon-hydrogen bond	Ser 720, Asp 855
			Pi- Sigma	Met 766
			Pi-Pi T-shaped	Phe 856
			Alkyl	Leu 718, Val 726, Ala 743, Leu 777
COX-2	C9	−7.3	Conventional Hydrogen Bond	Val 523
			Amide-Pi-Stacked	Gly 526
			Alkyl	Val 349, Leu 352, Trp 387, Ala 527
	C14	−7.2	Conventional Hydrogen Bond	Arg 513
			Carbon-hydrogen bond	His 90, Ser 353
			Alkyl	Val 349, Leu 352, Leu 359, Phe 518, Val 523, Ala 527, Leu 531
	C18	−7.4	Carbon-hydrogen bond	Ala 527, Ser 530
			Amide-Pi-Stacked	Gly 526
			Pi- Sulfur	Met 522
			Alkyl	His 90, Met 113, Val 116, Leu 117, Val 349, Leu 359, Ala 516, Val 523, Leu 531
	C19	−7.9	Pi- Sigma	Val 349
			Alkyl	Val 116, Val 523, Val 527, Leu 531, Leu 352
	C21	−7.2	Alkyl	Met 113, Val 116, Tyr 348, Val 349, Leu 352, Tyr 355, Leu 359, Trp 387, Phe 518, Met 522, Val 523, Ala 527, Leu 531
	C23	−7.8	Carbon-hydrogen bond	Ala 527, Ser 530
			Pi- Sulfur	Met 522
			Amide-Pi-Stacked	Gly 526
			Alkyl	His 90, Val 116, Val 349, Leu 352, Leu 359, Ala 516, Leu 531, Val 523
	Diclofenac	−7.8	Conventional Hydrogen Bond	Tyr 355
			Pi- Sigma	Val 349, Ala 527
			Amide-Pi-Stacked	Gly 526
			Alkyl	Leu 352, Leu 531
TNF-α	C8	−6.5	Conventional Hydrogen Bond	Gly 121
			Pi- Sigma	Tyr 59
			Alkyl	Leu 57, Tyr 119
	C9	−6.7	Conventional Hydrogen Bond	Gly 121
			Carbon-hydrogen bond	Tyr 59
			Alkyl	Leu 57, Tyr 119
	C18	−6.3	Carbon-hydrogen bond	Gly 121
			Alkyl	Leu 57, Tyr A 59, Tyr B 59,
	C19	−7.4	Pi-Pi-Stacked	Tyr 59
			Alkyl	Leu A 57, Leu 57, Tyr 59
	C23	−6.5	Carbon-hydrogen bond	Gly 121
			Pi- Sigma	Tyr 59
			Alkyl	Leu 57, Tyr 59, Tyr 119
	Diclofenac	−7.1	Conventional Hydrogen Bond	Leu 120
			Pi-Pi-Stacked	Tyr 59, Tyr 119

**FIGURE 7:**
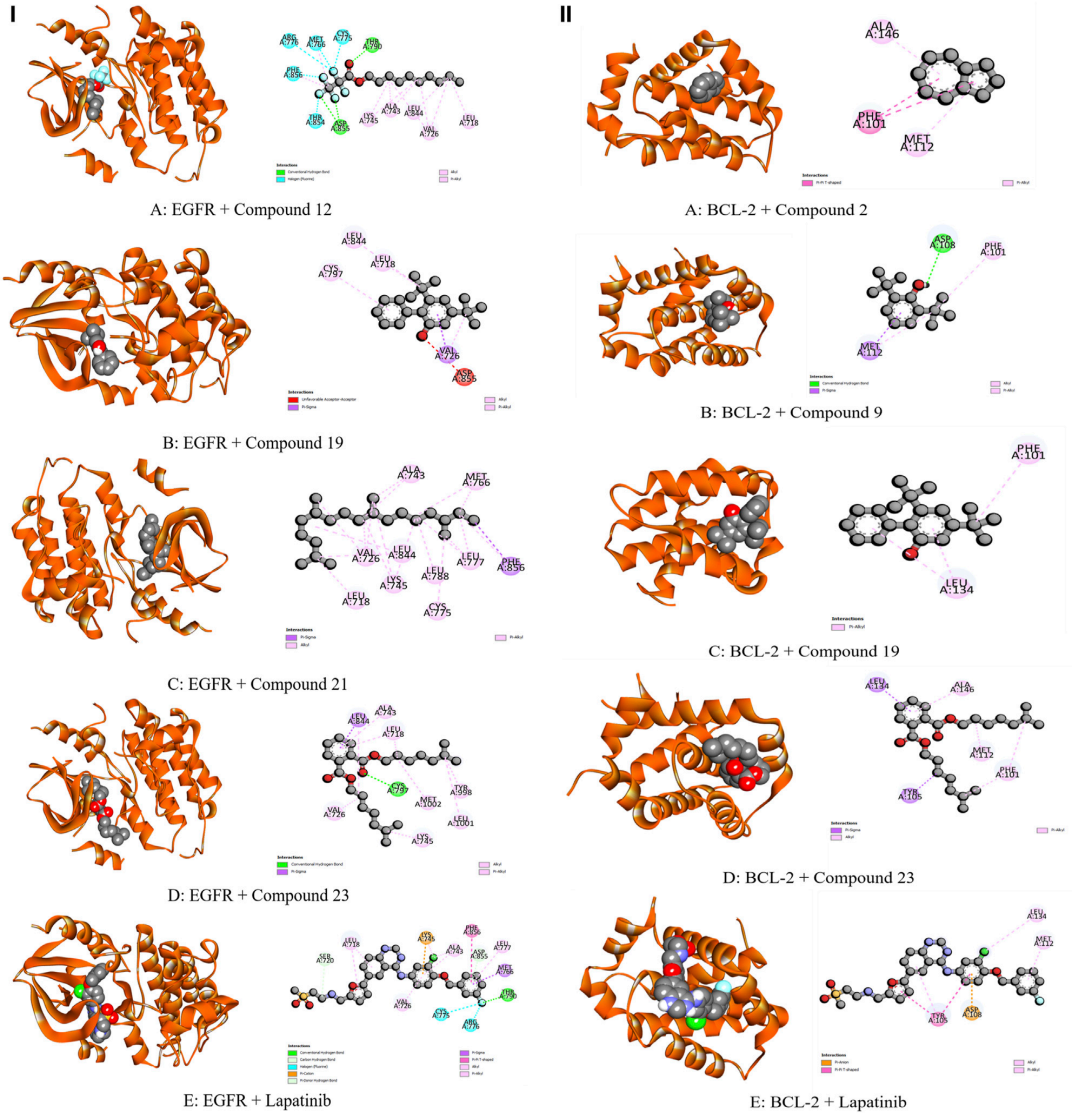
Molecular Interactions of Phytochemicals with EGFR and BCL-2 Enzymes: **(I)** Graphical representation of the molecular interactions of the most prominent phytocompounds with the EGFR enzyme with 3D visualization (Compound 12 = A, Compound 19 = B, Compound 21 = C, Compound 23 = D, and Standard Lapatinib = E). (II) Graphical representation of the molecular interactions of the most prominent phytocompounds with the BCL-2 enzyme with 3D visualization (Compound 2 = A, Compound 9 = B, Compound 19 = C, Compound 23 = D, and Standard Lapatinib = E).

EGFR, a pivotal regulator of cellular processes, undergoes conformational changes upon ligand binding, activating downstream pathways that promote cancer-related activities (Ongko et al., 2022). Elevated EGFR levels in gastric and breast cancers correlate with poor overall survival, advanced clinical stage, and resistance to therapy, emphasizing its prognostic relevance across various tumors. Additionally, in colorectal cancer, EGFR’s impact on tumor grade, stage, and survival underscores its significant role in cancer progression (Nicholson et al., 2001). In its interaction with EGFR, C19 formed one unfavorable acceptor-acceptor bond, one pi-sigma bond, and three alkyl bonds, yielding a binding affinity score of −7.8 kcal/mol. In contrast, C23 formed a solitary conventional hydrogen bond, a pi-sigma bond, and nine alkyl bonds, leading to a binding affinity of −7.4 kcal/mol. Additionally, C12 and C21 exhibited lower than −7 kcal/mol compared to the standard score of −10.6 kcal/mol (Table 7; Figure 7).

BCL-2, initially identified as the first anti-death gene, is implicated in cancer through various mechanisms such as chromosomal translocations, gene amplification, and altered expression regulation. Dysregulation of BCL-2 and related antiapoptotic proteins often contributes to chemotherapy resistance, making them potential targets for cancer therapy. Elevated BCL-2 expression is associated with poor prognosis in various cancers, emphasizing its significance in cancer development and treatment outcomes (Yip and Reed, 2008). Compound 19 exhibited the most excellent affinity for the BCL-2 receptor, establishing two pi-alkyl bonds. On the other hand, C23, which formed two pi-sigma and three alkyl bonds, demonstrated notable affinity with a score of −6.6 kcal/mol. In contrast, Lapatinib established three distinct types of bonds with the receptor, achieving a higher score of −8.4 kcal/mol. Furthermore, nine identified metabolites displayed binding affinities below −6 kcal/mol (Table 7; Figure 7).

**FIGURE 8:**
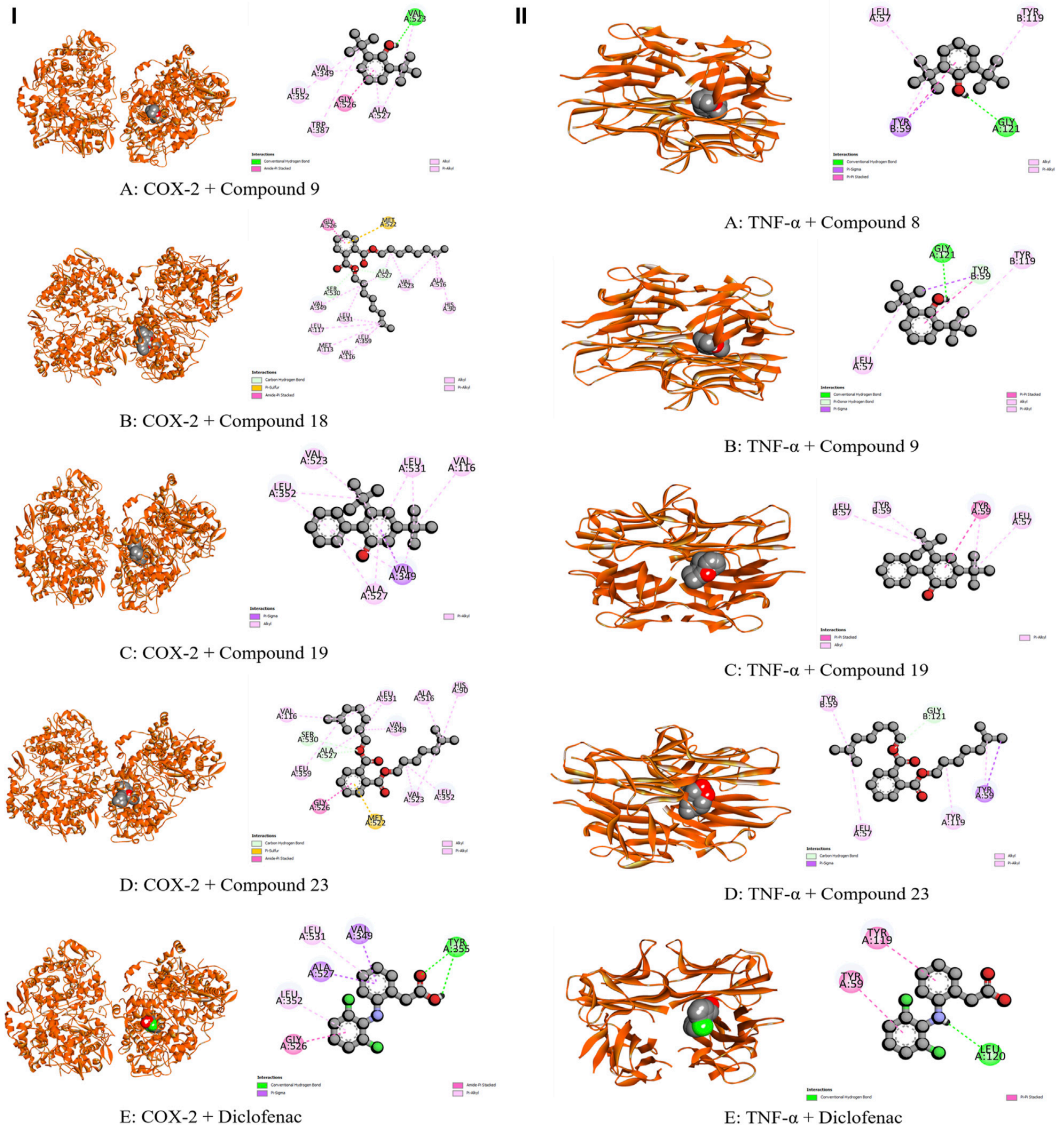
Molecular Interactions of Phytochemicals with COX-2 and TNF-α Enzymes: **(I)** Graphical representation of the molecular interactions of the most prominent phytocompounds with the COX-2 enzyme with 3D visualization (Compound 9 = A, Compound 18 = B, Compound 19 = C, Compound 23 = D, and Standard Diclofenac = E). **(II)** Graphical representation of the molecular interactions of the most prominent phytocompounds with the TNF-α enzyme with 3D visualization (Compound 8 = A, Compound 9 = B, Compound 29 = C, Compound 23 = D, and Standard Diclofenac = E).

Implicated in inflammation, cyclooxygenase-2 (COX-2) generates prostaglandins that play a pro-inflammatory role in the initial stages of the inflammatory process. However, recent studies propose a dual role for COX-2, suggesting that the prostaglandins it produces in later stages may contribute to the resolution of inflammation. Inhibition of COX-2 has been demonstrated to reduce inflammation in specific experimental models, emphasizing the complexity of its involvement in inflammatory responses (Williams et al., 1999). In our research, C19 and C23 showed remarkable affinity towards cox-2, where C19 formed a single pi-sigma and five alkyl bonds with the receptor, showcasing a score of −7.9 kcal/mol, while C23 bound with two carbon-hydrogen, one pi-sulfur, one amide-pi, and eight alkyl bonds with a score of −7.8 kcal/mol. However, with a score of −7.8 kcal/mol, Diclofenac interacted through one conventional hydrogen, two pi-sigma, one amide-pi, and two alkyl bonds. Considering the binding score, other metabolites, especially C9, C14, C18, and C21, scored lower than −7 kcal/mol by forming multiple interactions with the receptor (Table 7; Figure 8).

Infection or injury triggers inflammation as a physiological response, marked by acute inflammation characterized by cytokines and neutrophils. Chronic inflammation, which involves additional immune cells, is linked to various diseases, including cancer. In the context of inflammation, TNF-α plays a pivotal role by activating nuclear factor kappa B (NF-kB), leading to the expression of various inflammatory genes (Sethi et al., 2008). With remarkable binding affinity. In our study, C19 exhibited the highest efficacy against TNF- α, interacting with the receptor through one pi-pi and three alkyl bonds. Diclofenac (−7.1 kcal/mol) is bound with one conventional hydrogen bond and two pi-pi bonds. Notably, C8, C9, C18, and C23 significantly decreased docking scores compared to the standard diclofenac (Table 7; Figure 8). This suggests the potential for a potent anti-inflammatory effect from the crude extract of the plant.

Given the observed affinities towards various receptors, it is plausible to propose that the mentioned metabolites play a significant role by modulating the many biological impacts of the leaf extract. This highlights the possible significance of these chemicals in the overall pharmacological activity of the extract. Moreover, it is advisable to investigate these substances in future studies better to understand their distinct contributions and prospective therapeutic uses. Interestingly, C12, C18, and C23 exhibited significant binding efficacy with multiple receptors associated with particular disease conditions, highlighting their potential versatility across various disease states. However, computational ADME/T analysis of most active compounds towards those receptors is represented in Tables 8 and Table 9, where C2, C4, and C19 stand out for their excellent oral bioavailability as they only violate a single Rule of Five. Conversely, C18 and C23 present challenges by contravening three rules. However, most of the metabolites in Table 9, while violating two rules, still maintain a commendable level of oral bioavailability. Notably, C19 is the sole compound showing a potential for AMES toxicity, suggesting a need for caution due to potential carcinogenicity. On a positive note, all metabolites, except C4, exhibit negative hepatotoxicity, increasing their attractiveness as potential drug candidates.

**TABLE 8:** Absorption, Distribution, and Metabolism profile of the selected compounds that showed the best interaction against these receptors.

Properties	Absorption							Distribution				Metabolism						
Compouinds	Water solubility (log mol/L)	Caco2 permeability (log Papp in 10–6 cm/s)	Intestinal absorption (human) (% Absorbed)	Skin Permeability (log Kp)	P-glycoprotein substrate	P-glycoprotein I inhibitor	P-glycoprotein II inhibitor	VDss (human) (log L/kg)	Fraction unbound (human) (Fu)	BBB permeability	CNS permeability	CYP2D6 substrate	CYP3A4 substrate	CYP1A2 inhibitior	CYP2C19 inhibitior	CYP2C9 inhibitior	CYP2D6 inhibitior	CYP3A4 inhibitior
C2	−3.654	1.389	95.451	−1.649	No	No	No	0.519	0.441	0.77	−2.064	No	No	No	No	No	No	No
C4	−2.249	1.563	94.081	−1.518	No	No	No	0.523	0.383	0.525	−1.974	No	No	Yes	No	No	No	No
C8	−4.71	1.582	91.525	−2.156	No	No	No	0.749	0.004	0.461	−1.105	No	Yes	Yes	No	No	No	No
C9	−3.876	1.668	92.254	−2.364	No	No	No	0.545	0.042	0.47	−0.858	No	Yes	Yes	No	No	No	No
C12	−5.21	1.657	89.344	−2.168	No	Yes	No	0.038	0.237	0.587	−2.432	No	No	No	No	No	No	No
C14	−7.343	1.612	92.66	−2.719	No	No	Yes	0.272	0.028	0.767	−1.463	No	Yes	Yes	No	No	No	No
C18	−6.546	1.381	90.874	−2.672	No	Yes	Yes	0.35	0	−0.23	−2.329	No	Yes	No	Yes	No	No	No
C19	−5.143	1.773	93.479	−2.755	Yes	No	Yes	0.227	0	0.667	−0.794	No	Yes	Yes	Yes	Yes	No	No
C21	−8.773	1.424	92.38	−2.66	No	No	Yes	0.684	0	0.988	−1.265	No	Yes	Yes	No	No	No	No
C23	−6.757	1.425	91.448	−2.656	No	Yes	Yes	0.194	0	−0.184	−2.169	No	Yes	No	No	No	No	No
C37	−6.783	1.43	94.101	−1.197	No	No	No	0.565	0.145	0.855	−1.65	No	No	No	No	No	No	No
C45	−8.402	1.412	92.467	−2.36	No	No	No	0.667	0	0.946	−1.328	No	Yes	Yes	No	No	No	No

**TABLE 9:** Excretion, Toxicology and Drug-likeliness profile of the selected compounds that showed the best interaction against these receptors.

Properties	Excretion		Toxicity										Drug-likeness	
Compounds	Total Clearance (log mL/min/kg)	Renal OCT2 substrate	AMES toxicity	Max. Tolerated dose (human) (log mg/kg/day)	hERG I inhibitor	hERG II inhibitor	Oral rat acute toxicity (LD50) (mol/kg)	Oral rat chronic toxicity (LOAEL) (log mg/kg_bw/day)	Hepatotoxicity	Skin Sensitisation	T.Pyriformis toxicity (log ug/L)	Minnow toxicity (log mM)	Lipinski’s rule of five	Bioavailability score (%)
C2	0.208	No	No	0.507	No	No	1.66	2.271	No	Yes	0.347	1.425	No; 1 violation: MW < 250	0.55
C4	0.267	No	No	0.551	No	No	2.083	2.108	Yes	Yes	0.632	0.986	No; 1 violation: MW < 250	0.55
C8	0.805	No	No	0.493	No	No	2.171	1.484	No	Yes	1.435	0.126	No; 2 violations: MW < 250, XLOGP3>3.5	0.55
C9	0.781	No	No	0.409	No	No	2.346	1.736	No	Yes	1.667	−0.108	No; 2 violations: MW < 250, XLOGP3>3.5	0.55
C12	1.448	No	No	0.057	No	No	3.202	−0.064	No	Yes	2.314	−0.069	No; 2 violations: Rotors>7, XLOGP3>3.5	0.55
C14	2.032	No	No	−0.019	No	No	1.617	3.004	No	Yes	1.603	−1.6	No; 2 violations: Rotors>7, XLOGP3>3.5	0.55
C18	1.964	No	No	1.246	No	Yes	1.305	2.731	No	No	0.683	−3.04	No; 3 violations: MW > 350, Rotors>7, XLOGP3>3.5	0.55
C19	0.72	No	Yes	0.207	Yes	Yes	2.214	1.536	No	Yes	0.46	−1.964	No; 1 violation: XLOGP3>3.5	0.55
C21	1.644	No	No	0.19	No	Yes	1.556	1.169	No	Yes	1.333	−2.393	No; 2 violations: Rotors>7, XLOGP3>3.5	0.55
C23	1.652	No	No	1.112	No	Yes	1.249	2.695	No	No	0.664	−3.429	No; 3 violations: MW > 350, Rotors>7, XLOGP3>3.5	0.55
C37	1.472	No	No	0.545	No	No	1.589	1.417	No	Yes	2.076	−0.592	No; 2 violations: MW < 250, XLOGP3>3.5	0.55
C45	1.61	No	No	0.2	No	Yes	1.502	1.275	No	Yes	1.811	−1.703	No; 2 violations: Rotors>7, XLOGP3>3.5	0.55

## Conclusion

Upon phytochemical analysis and biological investigations of different fractionated extracts of *C. affinis*, the AQSF showed the most potent antioxidant, cytotoxic, and antimicrobial attributes among the other three. In contrast, the DCMSF exhibited the most potent antidiarrheal and analgesic activities among the others. The EASF also demonstrated notable activities in antioxidant, cytotoxicity, and antimicrobial tests. Upon GC-MS analysis of all four fractions, a total of 34 metabolites were found in AQSF, 20 metabolites in DCM, 7 metabolites in HSF, and 6 metabolites in EASF. At the same time, phytochemical screening showed the presence of different chemical classes. The obtained metabolites possess drug-like properties, which can account for the documented pharmacological actions. Computer-aided techniques also corroborate the role of these substances in producing these effects. Based on the *in-vitro*, *in-vivo*, GC-MS, and *in silico* observations of this study, it can be concluded that, the fractionated extracts, especially the aqueous soluble fraction (AQSF) of the vegetable *C. affinis* demand further extensive scientific research for the isolation of its phytochemicals and determination of their mode of action to employ medicinal actions along with their safety profiles.

## Data Availability

The raw data supporting the conclusions of this article will be made available by the authors, without undue reservation.

## Abbreviations

HSF, hexane soluble fraction; DCMSF, dichloromethane soluble fraction; EASF, ethyl acetate soluble fraction; AQSF, aqueous soluble fraction; BHT, butylated hydroxytoluene; VS, vincristine; ROS, reactive oxygen species; COX 1, cyclooxygenase-1; COX 2, cyclooxygenase-2; IC50, half maximal inhibitory concentration; LC50, lethal concentration 50; DPPH, 2,2-diphenyl-1-picryl-hydrazyl hydrate; GC-MS, Gas Chromatography-Mass Spectrometry; BCL-2, B-cell lymphoma 2; DHFR, Dihydrofolate reductase; DOR, Delta opioid receptors; EGFR, Epidermal growth factor receptor; GLR, Glutamate Receptors; KAS, ketoaryl-ACP synthase; KOR, kappa-opioid receptor; TNF-α, Tumor necrosis factor-alpha; URO, Urate oxidase.
